# Supplementary material for: Functionalizing tandem mass tags for streamlining click-based quantitative chemoproteomics
Source: Commun Chem. 2024 Apr 10;7:80. doi: 10.1038/s42004-024-01162-x (PMC11006884; doi:10.1038/s42004-024-01162-x)
Supplement: Supplementary file 2 — Supporting Information [file 42004_2024_1162_MOESM2_ESM.pdf]

Functionalizing tandem mass tags for streamlining click-based quantitative chemoproteomics

Nikolas R. Burton[1,2] and Keriann M. Backus[1,2,3,4,5,6#*]

1 Department of Biological Chemistry, David Geffen School of Medicine, UCLA, Los Angeles, California 90095, United States

2 Department of Chemistry and Biochemistry, UCLA, Los Angeles, California 90095, United States

3 Molecular Biology Institute, UCLA, Los Angeles, California 90095, United States

4 DOE Institute for Genomics and Proteomics, UCLA, Los Angeles, California 90095, United States

5 Eli and Edythe Broad Center of Regenerative Medicine and Stem Cell Research, UCLA, Los Angeles, California 90095, United States

6 Jonsson Comprehensive Cancer Center, UCLA, Los Angeles, California 90095, United States

* Corresponding Author: Keriann M. Backus, Biological Chemistry Department, David Geffen School of Medicine, UCLA, Los Angeles, CA, 90095, USA, E-mail: kbackus@mednet.ucla.edu

Table of Contents

(A) Supplementary Figures 2-11
(B) Supplementary Tables 11-12
Supplementary Methods:
(C) Chemistry Methods 12-16
(D) Biology Methods 17-21
(E) Mass Spectrometry Methods 21-25
(F) NMR Spectra 25-30
(G) Supplementary References 30-31

(A) Supplementary Figures

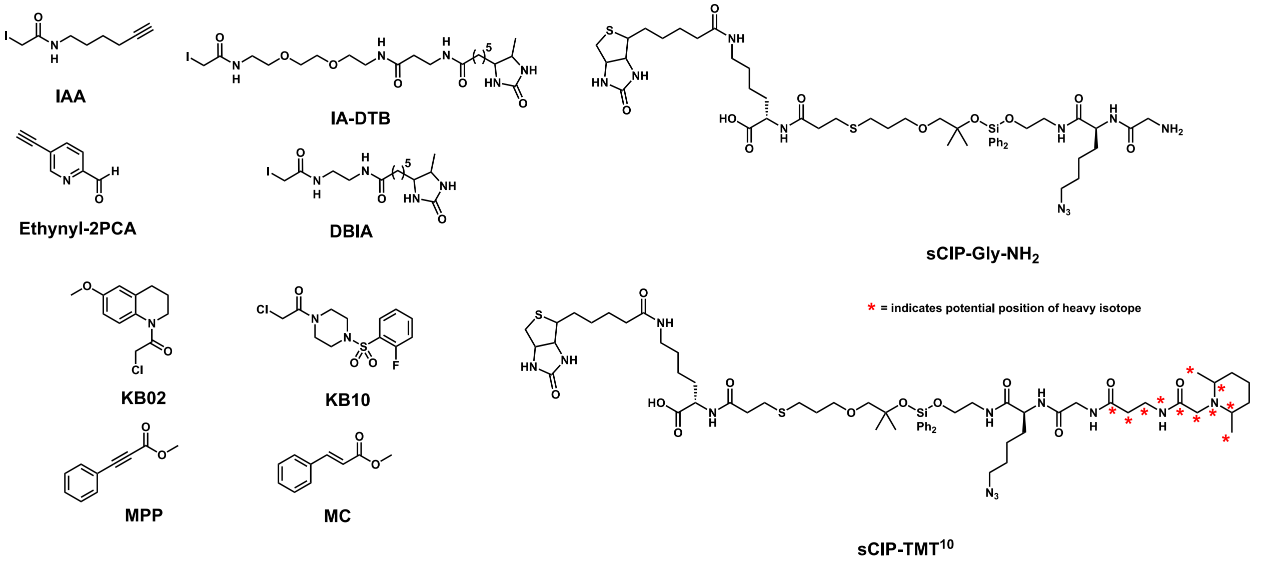

Figure S1. Structures of compounds used and referenced in this study.

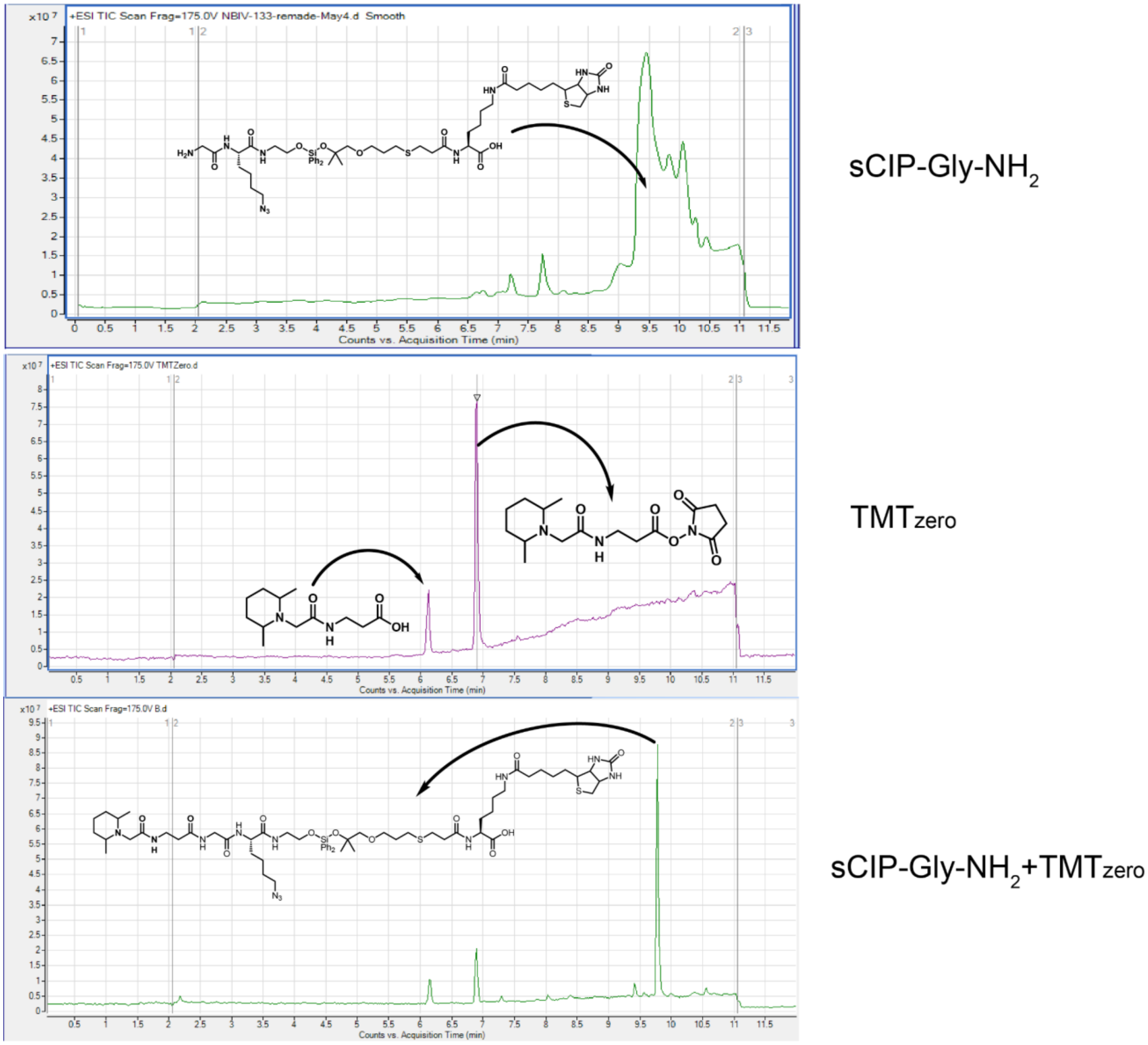

Figure S2. HPLC traces showing the sCIP-Gly-NH2 reagent, the TMTzero reagent, and the result of mixing these reagents 1:1 in DMSO:MeCN to form the sCIP-TMTzero reagent.

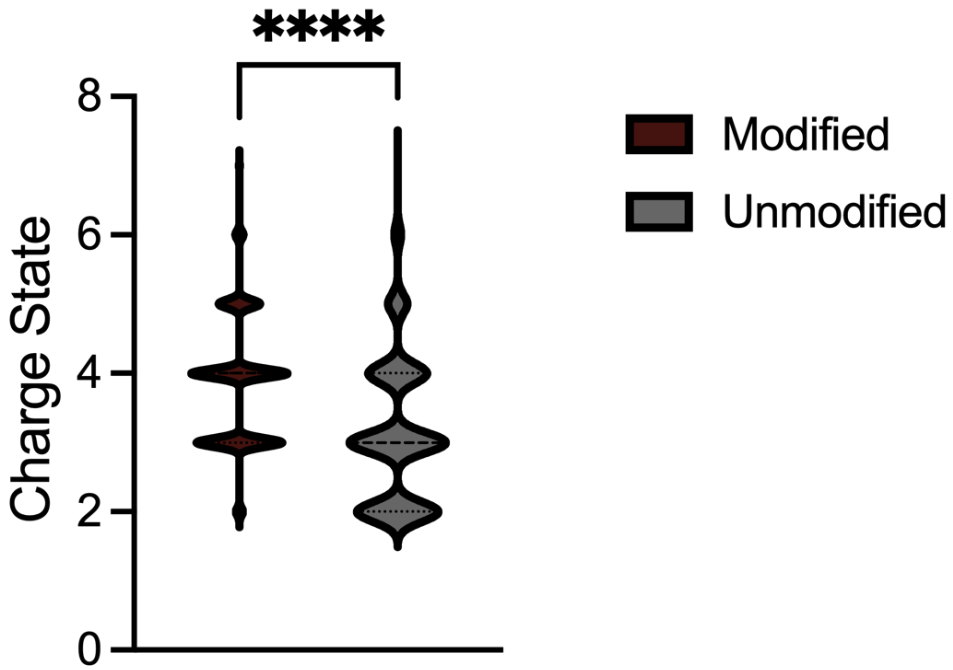

Figure S3. Comparison of charge state between peptides modified with sCIP-TMTzero and unmodified peptides. n=5 technical replicates. All MS data can be found in **Supplementary Data 1**.

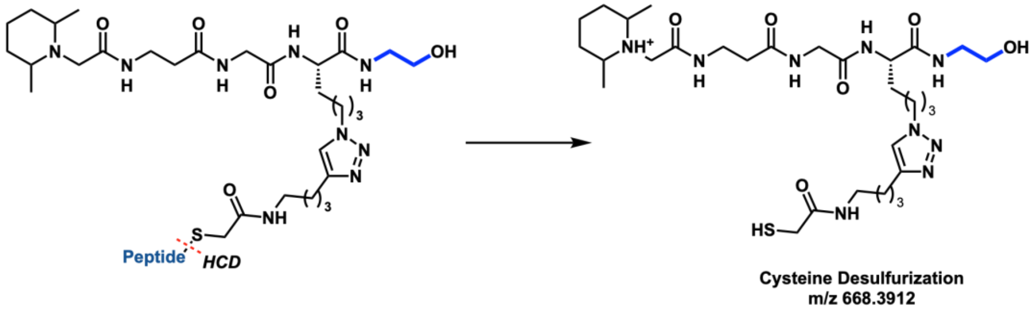

Figure S4. Alternative fragmentation of cysteine labeled peptides.

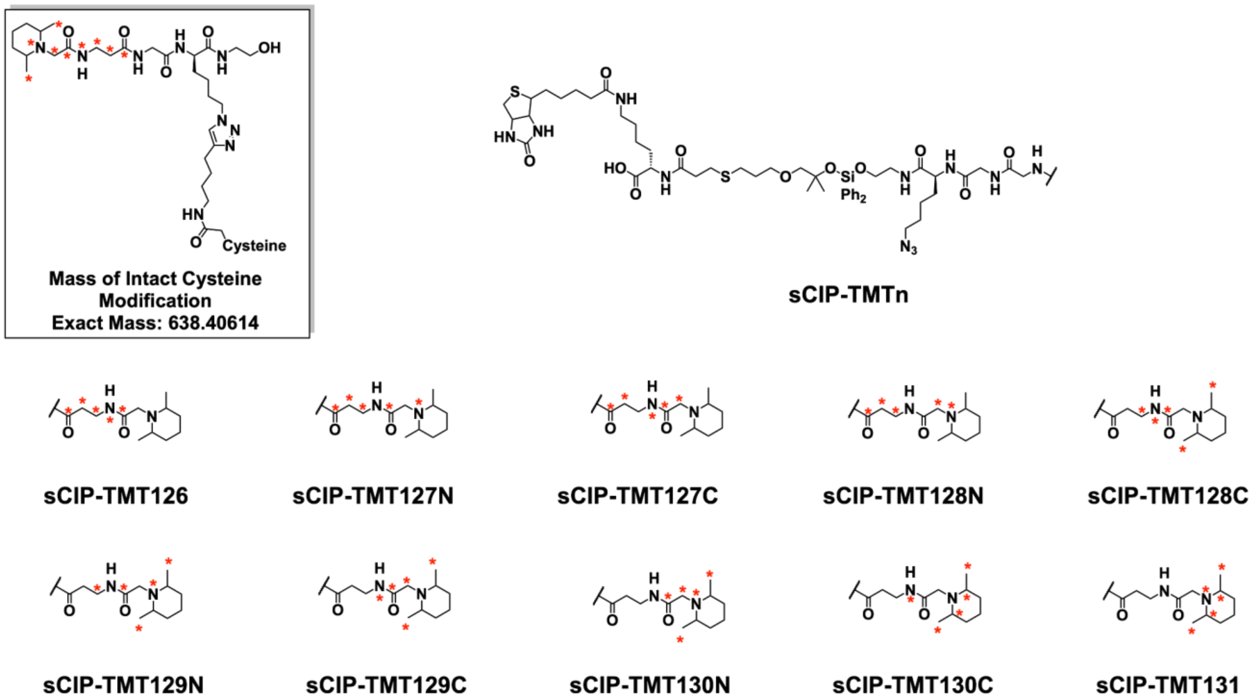

Figure S5. Structures of sCIP-TMT10 reagents formed in situ and used in figure 2 as well as intact modification mass. Red asterisk indicates site of heavy atom incorporation.

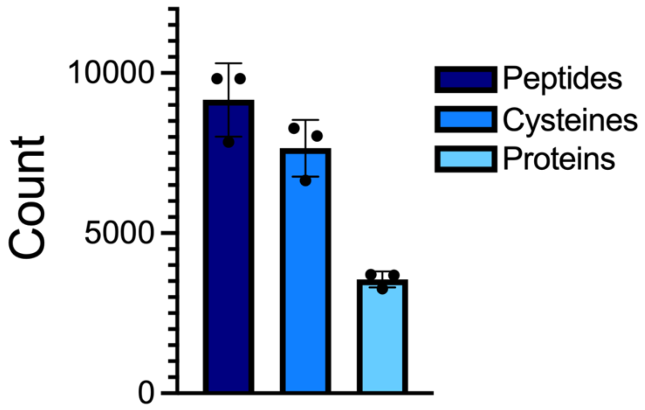

Figure S6. Peptides, cysteines, and protein counts for samples from **Figure 2C** mixed 1:5:10:15, acquired without FAIMS. Error bars on bar plots display standard deviation. n=3 biological replicates. All MS data can be found in **Table S3**.

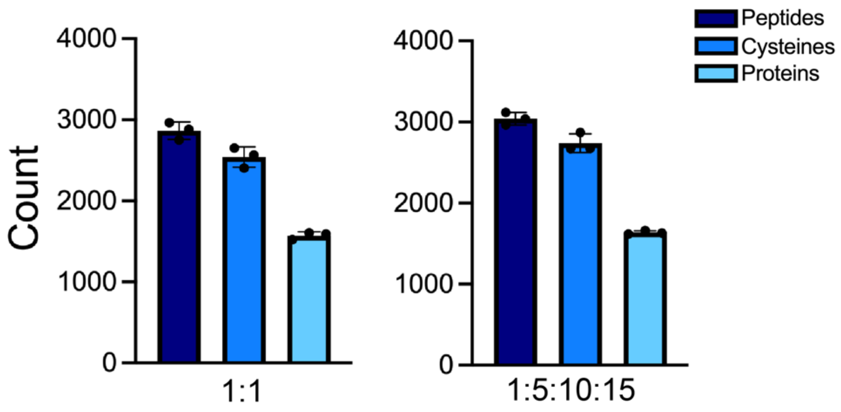

Figure S7. Peptides, cysteines, and protein counts for samples from **Figure 2D** mixed both 1:1 (left) and 1:5:10:15 (right), acquired with FAIMS. Error bars on bar plots display standard deviation. n=3 biological replicates. All MS data can be found in **Supplementary Data 2**.

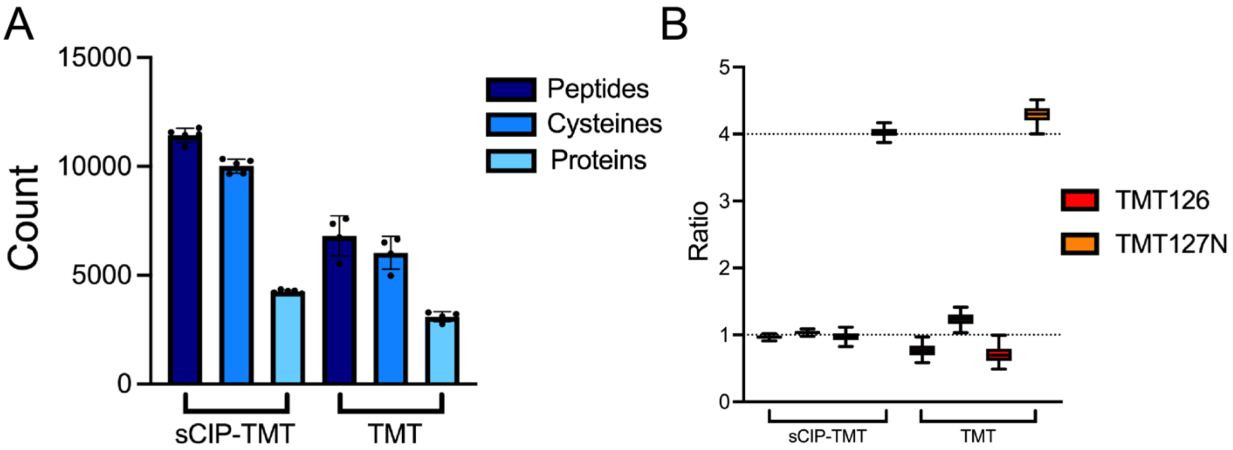

Figure S8. sCIP-TMT126 and sCIP-TMT127N labeled samples were combined either 1:1 or 1:4 directly after click, prior to sample preparation. IA-DTB labeled samples were taken through the sample preparation separately, enriched with streptavidin, then labeled with either TMT126 or TMT127N before being combined in 1:1 and 1:4 ratios. (A) Peptides, cysteines, and protein counts for samples analyzed using either sCIP-TMT analysis or TMT sample preparation. (B) Comparison of ratios for samples mixed in both 1:1 and 1:4 ratios using sCIP-TMT and TMT sample preparation. Box plots display 5th percentile, first quartile (Q1), median, third quartile (Q3), and 95th percentile values of the sample. Error bars on bar plots display standard deviation. For sCIP-TMT analysis n=5 biological replicates, and for TMT analysis n=4 biological replicates. All MS data can be found in **Supplementary Data 3**.

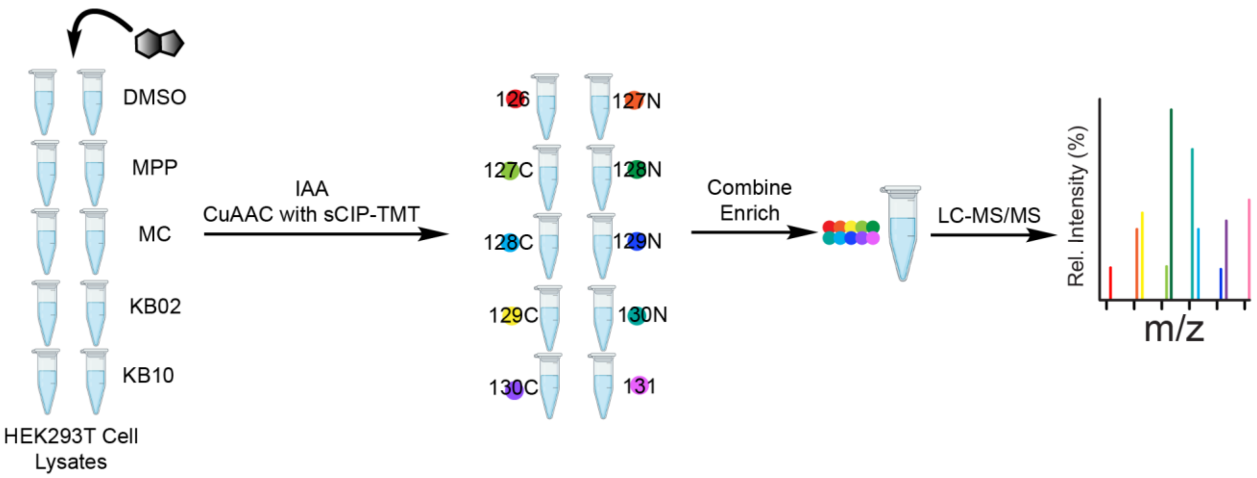

Figure S9. Workflow for electrophilic compound screening with sCIP-TMT used for **Figure 3**. Samples were prepared in triplicate with two channels per treatment group in each. DMSO treated samples were labeled with sCIP-TMT 126 and 127N; MPP with 127C and 128N; MC with 128C and 129N; KB02 with 129C and 130N; and KB10 with 130C and 131.

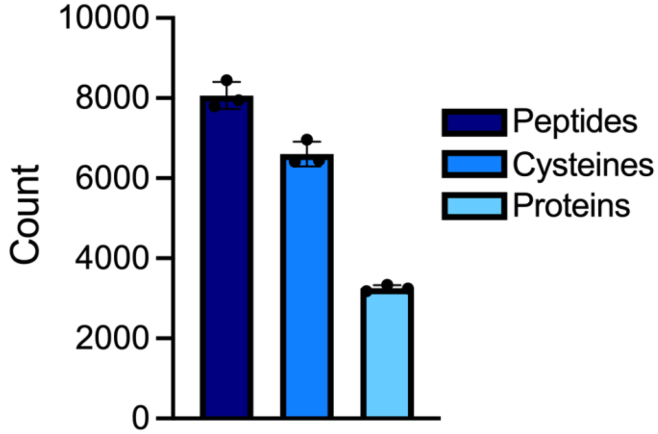

Figure S10. Coverage of Peptides, Cysteines and Proteins identified in our sCIP-TMT10 plex compound labeling experiment. Error bars on bar plots display standard deviation n=3 biological replicates. All MS data can be found in **Supplementary Data 4**.

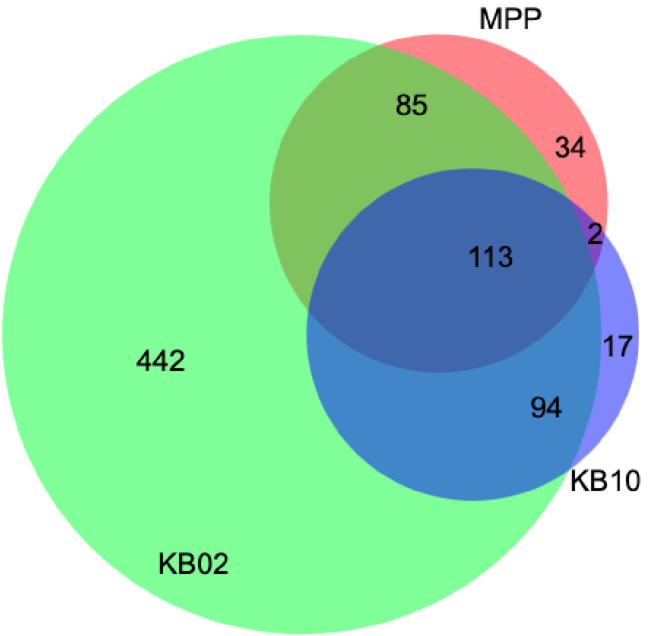

Figure S11. Venn Diagram comparing cysteines labeled (log_2 ratio>1) by **MPP**, **KB02**, and **KB10**. All MS data can be found in **Supplementary Data 4**.

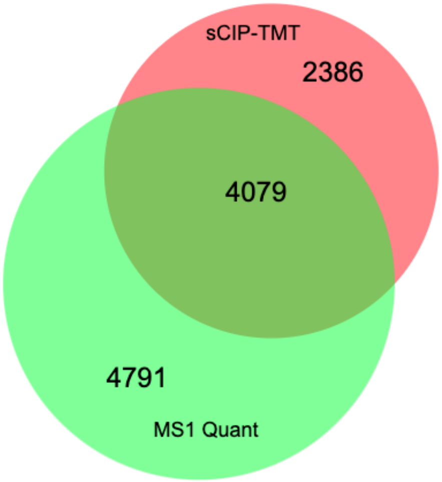

Figure S12. Venn Diagram comparing cysteines identified with sCIP-TMT and our previously described MS1 quantitation.[1] All MS data can be found in **Supplementary Data 4**.

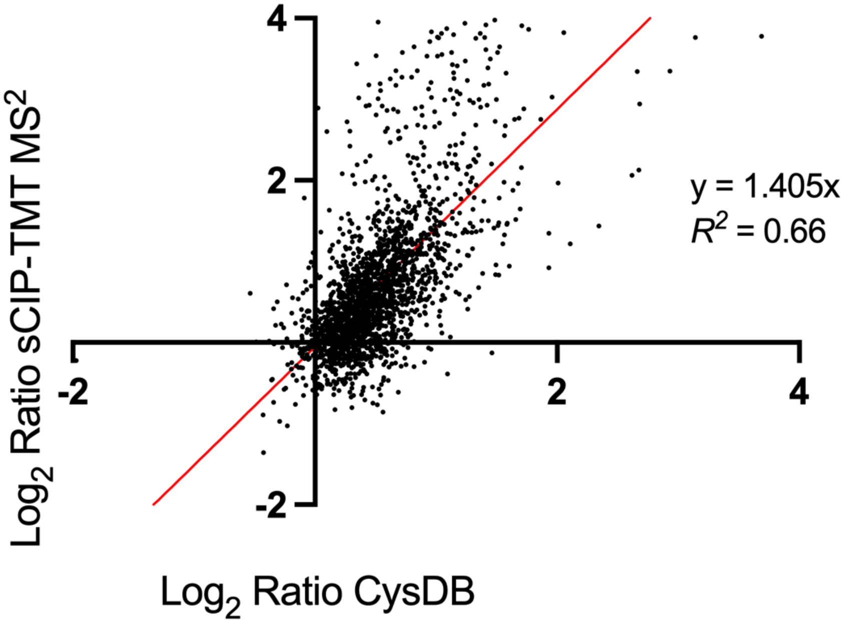

Figure S13. Comparison of the Log2 ratios for cysteines from CysDB (x-axis)[2] versus sCIP-TMT (y-axis) identified with scout fragment **KB02**. Linear regression performed y=1.405x, R^2 = 0.66. n=3 biological replicates. All MS data can be found in **Supplementary Data 4**.

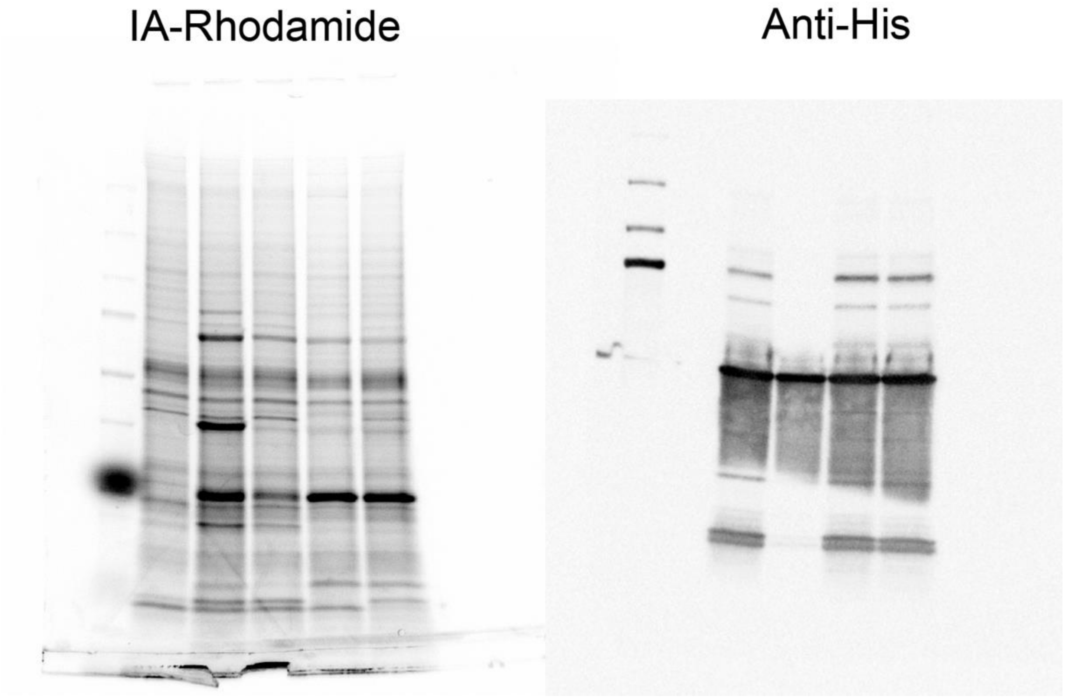

Figure S14. Full IA-rhodamine gel and anti-His western blot from **Figure 5F**.

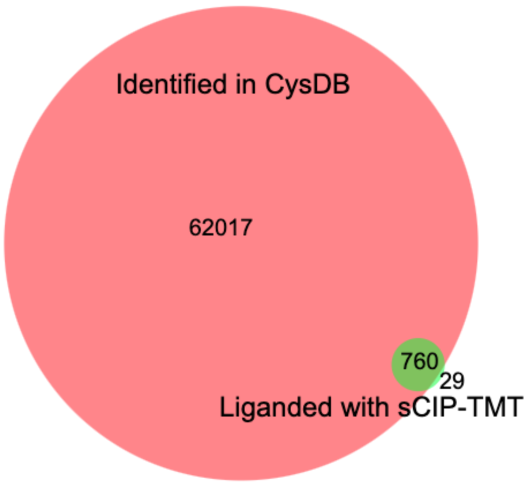

Figure S15. Venn Diagram comparing cysteines previously identified by CysDB and cysteines labeled (log_2 ratio>1) by one or more of the four electrophilic compounds used in this study. All MS data can be found in **Supplementary Data 4**.

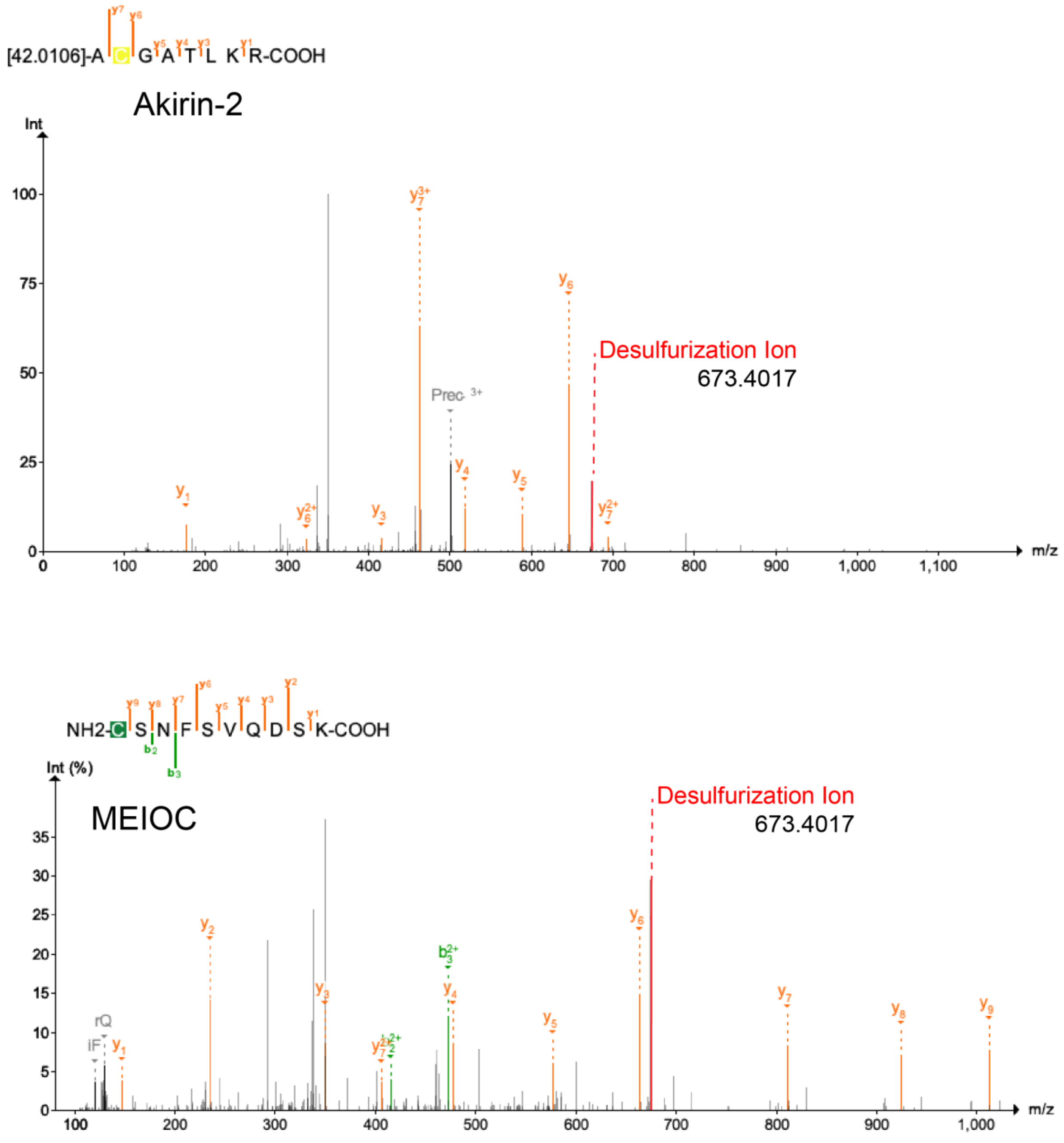

Figure S16. Identification of desulfurization ion in Akirin-2 and MEIOC peptide spectra.

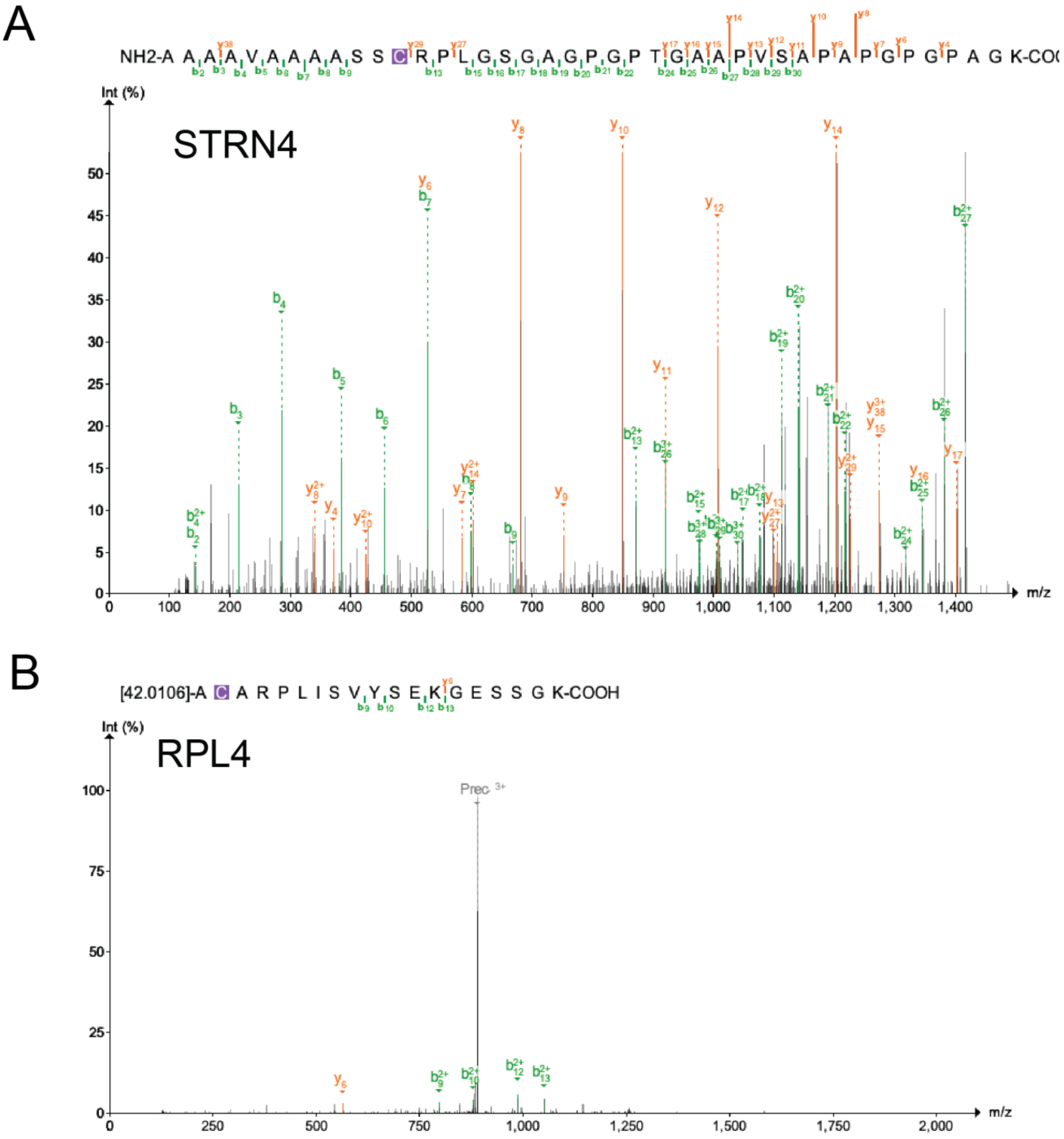

Figure S17. Peptide spectra of (A) Striatin-4 (STRN4) and (B) Large ribosomal subunit protein uL4 (RPL4) both lacking the desulfurization ion.

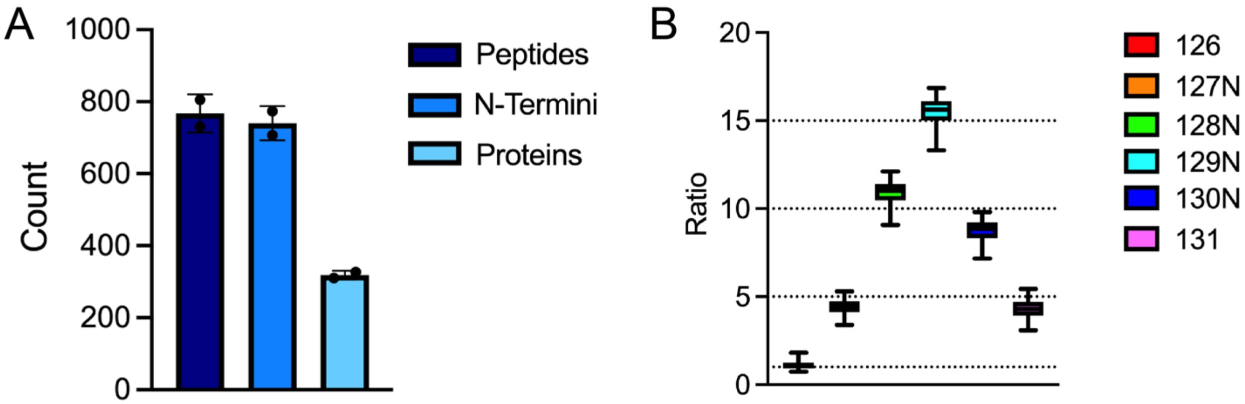

Figure S18. (A) Peptides, n-termini, and protein counts for samples analyzed using ethynyl-2PCA labeling and sCIP-TMT sample preparation. (B) Ratios for samples labeled with the indicated sCIP-TMT reagent and mixed in a 1:5:10:15:10:5 ratio. Box plots display 5th percentile, first quartile (Q1), median, third quartile (Q3), and 95th percentile values of the sample. Error bars on bar plots display standard deviation. n=2 biological replicates. All MS data can be found in All MS data can be found in **Supplementary Data 2**.

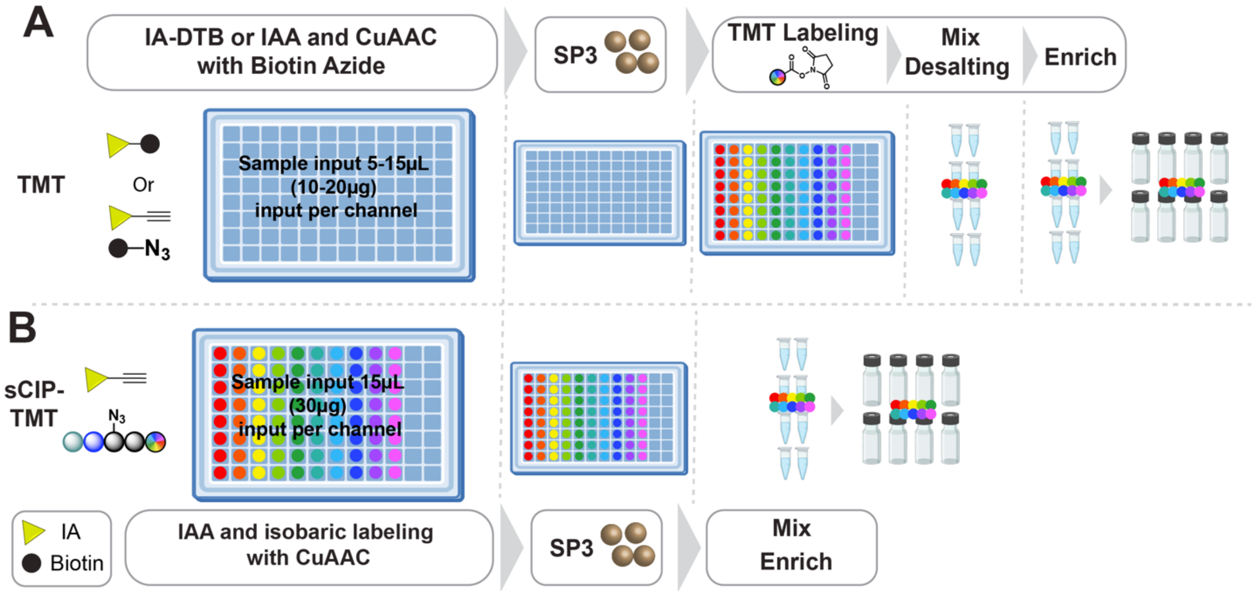

Figure S19. Comparison of (A) TMT sample prep workflow and (B) hypothesized sCIP-TMT sample preparation workflow in a 96-well format to allow for automation-compatibility.

(B) Supplemental Tables

Table S1. Mixing ratios of sCIP-TMT[10] reagents for Figure 3

sCIP-TMT[10] Reagent	Ratio
126	1
127N	5
127C	10
128N	5
128C	15
129N	1
129C	10
130N	5
130C	15
131	5

Supplementary Methods

(C) Chemistry Methods

General Methods. All reactions were performed in dried glassware under an atmosphere of dry N_2 unless otherwise stated. Silica gel P60 (SiliCycle) was used for column chromatography. Plates were visualized by fluorescence quenching under UV light or by staining with iodine, $KMnO_4$, or bromocresol green. Other reagents were purchased from Sigma-Aldrich (St. Louis, MO), Alfa Aesar (Ward Hill, MA), EMD Millipore (Billerica, MA), Fisher Scientific (Hampton, NH), Oakwood Chemical (West Columbia, SC), Combi-blocks (San Diego, CA) and Cayman Chemical (Ann Arbor, MI) and used without further purification. Additionally, all isotopically enriched reagents were purchase from Sigma-Aldrich (St. Louis, MO) or Cambridge Isotope Laboratories (Cambridge, MA) and used without further purification. ^{1}H NMR and ^{13}C NMR spectra for characterization of new compounds and monitoring reactions were collected in $CDCl_3$, CD_3OD, D_2O or DMSO-d_6 (Cambridge Isotope Laboratories, Cambridge, MA) on a Bruker AV 500 MHz spectrometer or Bruker AV 400 MHz in the Department of Chemistry & Biochemistry at The University of California, Los Angeles. All chemical shifts are reported in the standard notation of parts per million using the peak of residual proton signals of the deuterated solvent as an internal reference. Coupling constant units are in Hertz (Hz). Splitting patterns are indicated as follows: br, broad; s, singlet; d, doublet; t, triplet; q, quartet; m, multiplet; dd, doublet of doublets; dt, doublet of triplets. Low-resolution mass spectrometry was performed on an Agilent Technologies InfinityLab LC/MSD single quadrupole LC/MS (ESI source). High-resolution mass spectrometry was performed on a Waters LCT Premier with ACQUITY LC and autosampler (ESI source). Cell

culture reagents including Dulbecco's phosphate-buffered saline (DPBS), Dulbecco's modified Eagle's medium (DMEM)/high glucose media, Roswell Park Memorial Institute (RPMI) media, trypsin-EDTA and penicillin/streptomycin (Pen/Strep) were purchased from Fisher Scientific. All protein concentrations were determined using a Bio-Rad DC protein assay kit using reagents from Bio-Rad Life Science (Hercules, CA).

All DADPS reagents were prepared as previously described with procedures recounted here for ease of reference.[1]

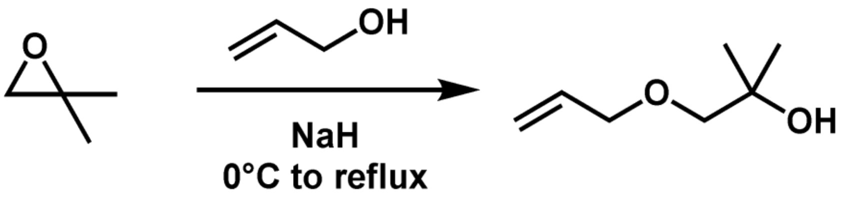

1-(allyloxy)-2-methylpropan-2-ol
To a 100 mL round-bottom flask was added allyl alcohol (10.2 g, 12.0 mL, 3 Eq, 176 mmol) and cooled to 0°C. To this solution was slowly added sodium hydride (2.4 g, 60% Wt, 1.0 Eq, 58.8 mmol) and then let stir at 0°C for 20 min. Next, 2,2-dimethyloxirane (4.24 g, 5.22 mL, 1 Eq, 58.8 mmol) was added and solution refluxed at 52°C. Upon completion of reaction as determined by TLC (3 hours) the reaction mixture was diluted with sat. NH_4Cl and extracted with Et_2O (3x 30mL). Combined organic layers were dried over sodium sulfate and volatiles removed under reduced pressure. The crude residue was purified by vacuum distillation yielding the desired alcohol as a clear liquid (5.18g, 67.7%). All analyses were consistent with previously reported data.[3]
^{1}H NMR (400 MHz, $CDCl_3$) δ 5.96 – 5.82 (m, 1H), 5.30 – 5.14 (m, 2H), 4.02 (ddt, J = 5.1, 3.2, 1.4 Hz, 2H), 3.26 (d, J = 3.1 Hz, 2H), 1.20 (d, J = 3.4 Hz, 6H).

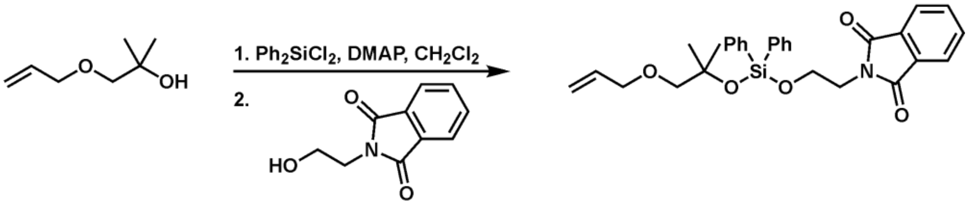

2-(6,6-dimethyl-4,4-diphenyl-3,5,8-trioxa-4-silaundec-10-en-1-yl)isoindoline-1,3-dione
To an oven dried 250mL round-bottom flask was added DMAP (2.534 g, 2.25 Eq, 20.74 mmol), 1-(allyloxy)-2-methylpropan-2-ol (1.200 g mg, 1 Eq, 9.217 mmol) and capped with septa. The system was purged with argon and dry CH_2Cl_2 (32 mL) was added. The solution was cooled to 0°C followed by dropwise addition of diphenyldichlorosilane (2.917 g, 2.37 mL, 1.25 Eq, 11.52 mmol). The solution was then allowed to warm to room temperature. After 5h the first addition was determined complete by TLC. The solution was cooled to 0°C and N-(2-Hydroxyethyl) phthalimide (1.85 g, 1.05 Eq, 9.68 mmol) was added. The solution was allowed to slowly warm to ambient temperature and stir overnight. Upon completion the reaction was diluted with sat. sodium bicarbonate and extracted with CH_2Cl_2 (3x 20mL). Combined organic layers were washed with brine and dried over sodium sulfate. Crude product was purified by silica column chromatography (1:9 to 1:3 ethyl acetate:hexanes) to yield the pure product as a clear oil (3.25 g, 70%).
^{1}H NMR (400 MHz, $CDCl_3$) δ 7.83 – 7.76 (m, 2H), 7.73 – 7.66 (m, 2H), 7.62 – 7.50 (m, 5H), 7.35 – 7.28 (m, 2H), 7.25 – 7.18 (m, 3H), 5.83 (ddt, J = 17.3, 10.7, 5.5 Hz, 1H), 5.24 – 5.09 (m, 2H),

4.02 – 3.97 (m, 2H), 3.92 – 3.86 (m, 4H), 3.26 (s, 2H), 1.23 (s, 6H). All analyses were consistent with previously reported data.[1]

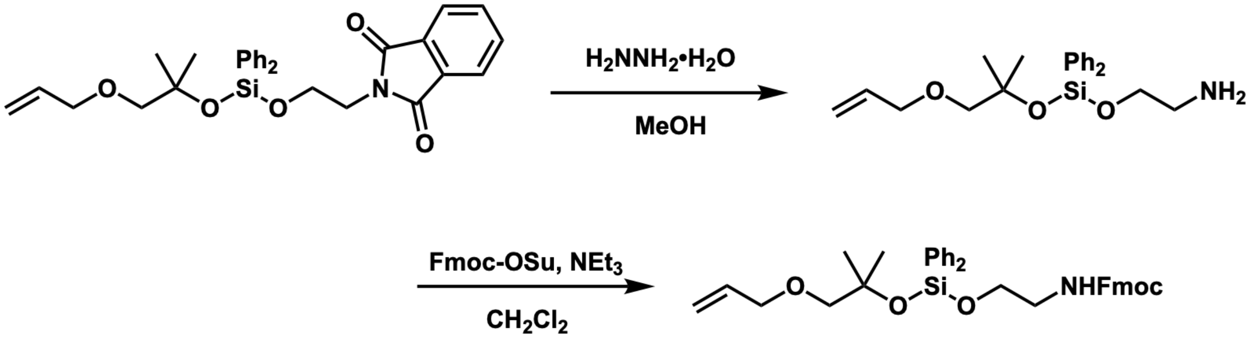

(9H-fluoren-9-yl)methyl (6,6-dimethyl-4,4-diphenyl-3,5,8-trioxa-4-silaundec-10-en-1-yl)carbamate

To an oven dried 250mL round-bottom flask was added 2-(6,6-dimethyl-4,4-diphenyl-3,5,8-trioxa-4-silaundec-10-en-1-yl)isoindoline-1,3-dione (2.64 g, 1 Eq, 5.26 mmol). The vial was capped and purged with nitrogen followed by addition of MeOH (26.3mL) and then dropwise addition of hydrazine hydrate (1.05 g, 1.02 mL, 4 Eq, 21.1 mmol). Solution let stir at ambient temperature overnight. Upon completion, reaction mixture diluted with 1M sodium carbonate and 1M oxalic acid and extracted with ethyl acetate (3x 30mL). Then, combined organic extracts washed with brine and dried over sodium sulfate. Volatiles were removed under reduced pressure and material used in the next step without further purification

To an oven dried 250 mL round-bottom flask was added Fmoc-osu (2.13 g, 1.2 Eq, 6.32 mmol), capped, and purged with argon. The crude amine from the first step was dissolved in dry CH_2Cl_2 (26 mL) and added to this flask. Reaction mixture was then cooled to 0°C and triethylamine (1.28 g, 1.76 mL, 2.4 Eq, 12.6mmol) added. The solution was allowed to stir at room temperature for 16 hours. Upon completion, the reaction was diluted with water and extracted with CH_2Cl_2 (3x30mL). Organic layers combined and washed with brine then dried over sodium sulfate. The crude material was purified by silica column chromatography (1:9 to 1:3 ethyl acetate:hexanes) to yield the desired product as a pale yellow oil (1.89g, 61%).

^{1}H NMR (400 MHz, $CDCl_3$) δ 7.80 (d, J = 7.6 Hz, 2H), 7.69 (dt, J = 6.7, 1.5 Hz, 4H), 7.67 – 7.63 (m, 2H), 7.47 – 7.29 (m, 10H), 5.87 (ddt, J = 16.3, 10.7, 5.6 Hz, 1H), 5.56 (d, J = 5.9 Hz, 1H), 5.29 – 5.12 (m, 2H), 4.42 (d, J = 6.9 Hz, 2H), 4.25 (t, J = 6.9 Hz, 1H), 3.94 – 3.85 (m, 4H), 3.41 (q, J = 5.3 Hz, 2H), 3.30 (s, 2H), 1.32 (s, 6H). All analyses were consistent with previously reported data.[1]

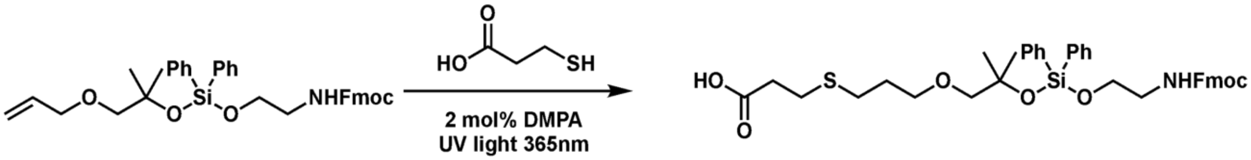

1-(9H-fluoren-9-yl)-10,10-dimethyl-3-oxo-8,8-diphenyl-2,7,9,12-tetraoxa-16-thia-4-aza-8-silanonadecan-19-oic acid

To an oven dried pressure tube was added (9H-fluoren-9-yl)methyl (6,6-dimethyl-4,4-diphenyl-3,5,8-trioxa-4-silaundec-10-en-1-yl)carbamate (494 mg, 1 Eq, 832 µmol), 3-mercaptopropionic acid (88.3 mg, 72.4 µL, 1 Eq, 832 µmol), and DMPA (4.26 mg, 0.02 Eq, 16.6 µmol). The vial was placed under a nitrogen atmosphere through vacuum purge cycles (3 cycles) and then the vial was capped. The vial was then irradiated using UV light (365nm, 4W compact lamp) with slow stirring and the whole setup was wrapped in aluminum foil. After 24 hours, full conversion was observed by NMR. The crude mixture was then dissolved in ethyl acetate and washed with sat.

sodium bicarbonate (3x 5mL), sat. ammonium chloride (1x 5mL), and brine. The organic layer was then dried over sodium sulfate and concentrated under reduced pressure to yield the desired product as a pale yellow wax (541mg, 93%).
^{1}H NMR (400 MHz, $CDCl_3$) δ 7.78 (d, *J* = 7.6 Hz, 2H), 7.64 (dd, *J* = 19.6, 4.5 Hz, 6H), 7.37 (ddt, *J* = 25.2, 14.3, 5.2 Hz, 10H), 4.41 (d, *J* = 6.9 Hz, 2H), 4.23 (t, *J* = 6.8 Hz, 1H), 3.90 – 3.69 (m, 2H), 3.46 – 3.16 (m, 6H), 2.73 (t, *J* = 7.2 Hz, 2H), 2.57 (dt, *J* = 22.3, 7.3 Hz, 4H), 1.77 (t, *J* = 6.9 Hz, 1H), 1.27 (m, 8H). All analyses were consistent with previously reported data.[1]

General Procedure for solid-phase synthesis of peptides:

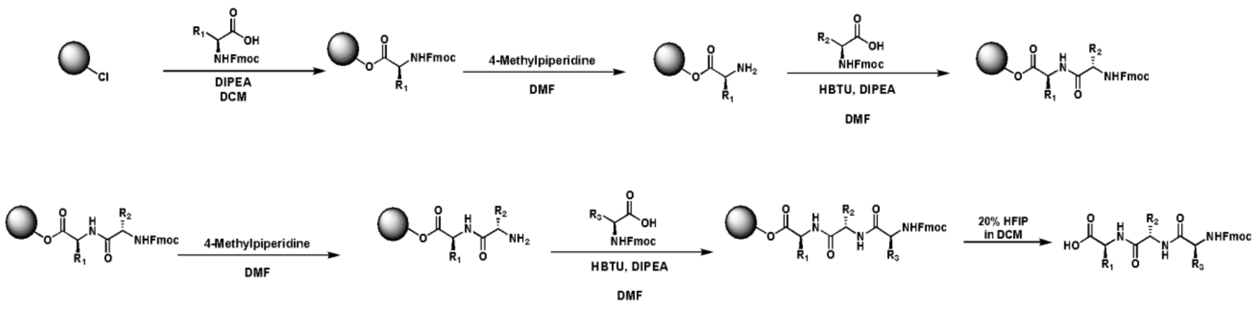

Peptides were manually synthesized according to the following general procedure.

Loading of the 2-chlorotrityl chloride resin: 2-chlorotrityl chloride resin (100-200 mesh, 0.1-0.9mmol/g) was added to a solid-phase vessel and swelled in dry CH_2Cl_2 for 1 hr. The CH_2Cl_2 was vacuum filtered off and first fmoc protected amino acid (2 Eq) was dissolved in dry CH_2Cl_2 and diisopropylethylamine (DIPEA) (3Eq.) and loaded onto resin. This was left to incubate for 1hr, after which the solution was vacuum filtered off and resin washed thoroughly with CH_2Cl_2, DMF, and MeOH. In the case of N-Fmoc-Biotin-Lys, the loading step was performed in DMF.

Amino Acid Coupling: Coupling of standard amino acids was carried out through treatment of the deprotected resin with 3 equivalents of Fmoc-protected amino acids, 3 equivalents of *N,N,N′,N′*-Tetramethyl-*O*-(1*H*-benzotriazol-1-yl)uronium hexafluorophosphate (HBTU), and 6 equivalents of DIPEA in DMF for 30 min. Each coupling was performed twice unless the amino acid used was valuable in which case the coupling was left longer. In between coupling and deprotection steps the resin was washed thoroughly with CH_2Cl_2, then DMF, MeOH, and CH_2Cl_2. Coupling of compounds to resin was monitored using the Kaiser test.

Fmoc Deprotection: Removal of *N*-terminal Fmoc protecting groups was carried out by treating the resin with 50% 4-methylpiperidine in DMF (3 x 1 min). After deprotection the resin was washed thoroughly with CH_2Cl_2, then DMF and CH_2Cl_2. Complete deprotection was monitored using the Kaiser test.

Resin Cleavage: Fully assembled peptides were then thoroughly washed with CH_2Cl_2 and the dried resin was incubated with 20% hexafluoroisopropanol (HFIP) for 10 minutes. The resulting solution was collected into a round-bottom flask and the process was repeated two times. The collected peptide solution was concentrated down to 2-3 mL and precipitated into cold diethyl ether. The ether was decanted off and the peptide dissolved in water. The desired peptides were obtained as fluffy white-pale yellow solids after lyophilization. LC-MS analysis revealed only minor impurities and tags were used without further purification.

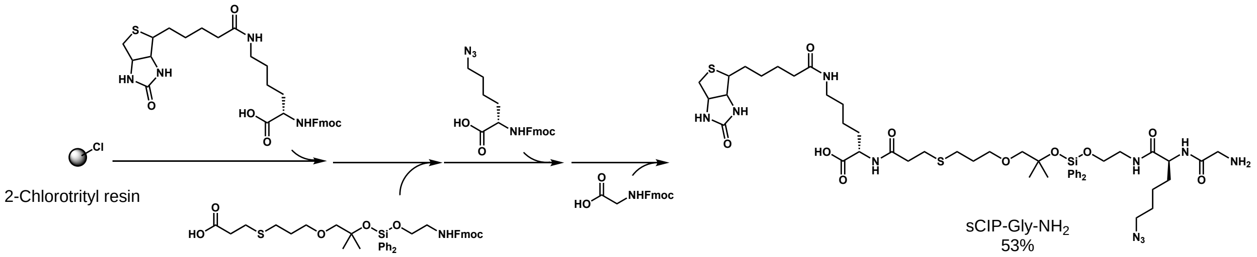

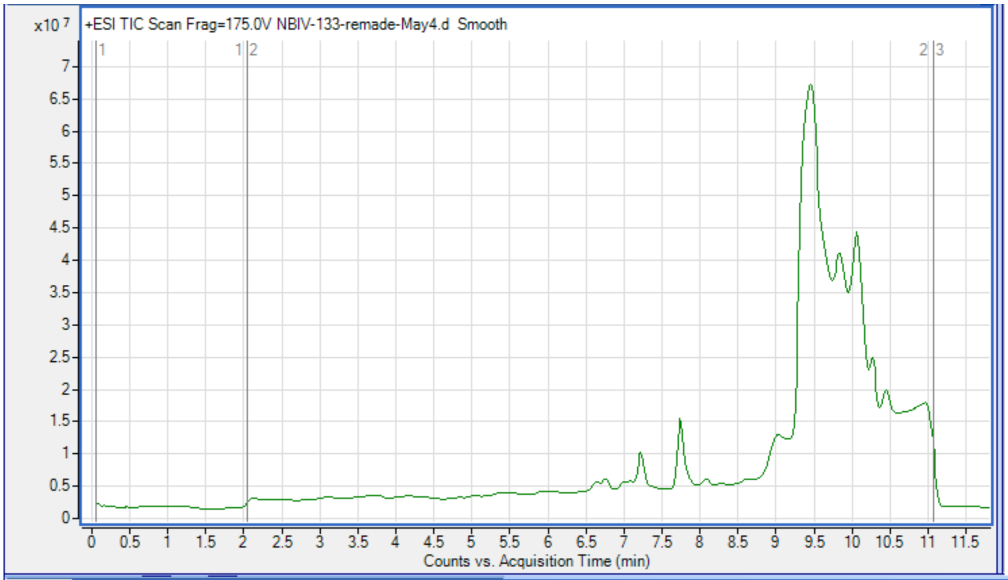

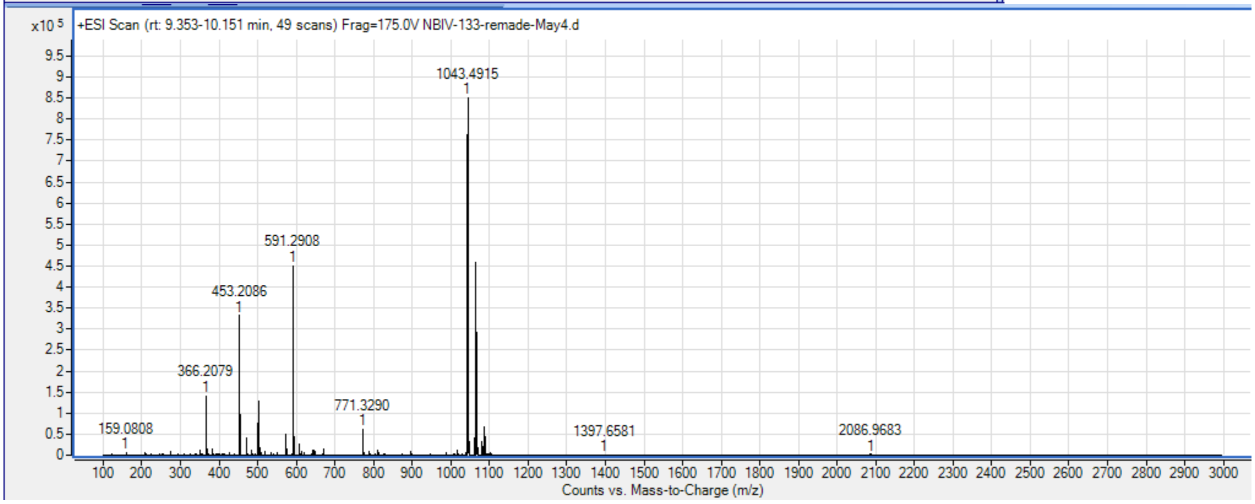

HRMS (ESI-MS) *m/z*: Calculated $[M+H]^+$ for $C_{48}H_{74}N_{10}O_{10}S_2Si$ = 1043.4872 , Found $[M+H]^+$ = 1043.4915

(D) Biology Methods

Cell culture and preparation of cell lysates.

Cell culture reagents including Dulbecco's phosphate-buffered saline (DPBS), Dulbecco's modified Eagle's medium (DMEM)/high glucose media, trypsin-EDTA, dialyzed Fetal Bovine Serum (FBS), and penicillin/streptomycin (Pen/Strep) were purchased from Fisher Scientific. Fetal Bovine Serum (FBS) were purchased from Avantor Seradigm (lot # 214B17). All cell lines were obtained from ATCC and were maintained at a low passage number (< 20 passages). HEK293T (ATCC: CRL-3216) cells were cultured in DMEM supplemented with 10% FBS and 1% antibiotics (Penn/Strep, 100 U/mL). Media was filtered (0.22 μm) prior to use. Cells were maintained in a humidified incubator at 37 °C with 5% CO2. Cell lines were tested for mycoplasma using the Mycoplasma Detection Kit (InvivoGen). Cells were harvested by centrifugation (4,500 g, 5 min, 4 °C), washed twice with cold PBS, resuspended in PBS, sonicated, and clarified by centrifuging (3,000 g, 5 min, 4 °C). The lysates were then transferred to a new microcentrifuge tube. Protein concentrations were determined using a Bio-Rad DC protein assay kit from Bio-Rad Life Science (Hercules, CA) and the lysate diluted to the working concentrations indicated below.

Preparation of sCIP-TMT capture reagents for CuAAC.
Each TMT channel (29mM in MeCN) was mixed in an equimolar ratio with sCIP-Gly-NH2 (29mM in DMSO) in a 1.5mL centrifugal tube and let react for 1h at ambient temperature. After 1h, 0.5 equivalents of hydroxylamine (10mM in DMSO) was added and let react for 15 minutes after which the sCIP-TMT conjugate was ready to be used for CuAAC.
<u>Note: if small amounts of volume are stuck on the side of tubes it is best to briefly centrifuge the sample to allow for complete mixing.</u>

Proteomic sample preparation for sCIP-TMT profiling of Cysteines.
HEK293T proteome (100 μL of 2 mg/mL) in a 1.5mL centrifugal tube was first labeled with **IAA** (1 μL of 50 mM stock solution in DMSO, final concentration = 500 μM) for 1h at ambient temperature. CuAAC was performed with the pre-formed sCIP-TMT capture reagent (final concentration = 1 mM), TCEP (2 μL of fresh 50 mM stock in water, final concentration = 1 mM), TBTA (6 μL of 1.7 mM stock in DMSO/t-butanol 1:4, final concentration = 100 μM), CuSO4 (2 μL of 50 mM stock in water, final concentration = 1 mM), and 0.2% SDS for 1h at ambient temperature. After CuAAC labeling, each sample was treated with 0.5 μL benzonase (Fisher Scientific, 70- 664-3) for 30 min at 37 °C. For each 100 μL sample (1 mg/mL protein concentration), 20 μL Sera-Mag SpeedBeads Carboxyl Magnetic Beads, hydrophobic (GE Healthcare, 65152105050250) and 20 μL Sera-Mag SpeedBeads Carboxyl Magnetic Beads, hydrophilic (GE Healthcare, 45152105050250) were mixed and washed with water for three times. The bead slurries were then transferred to the CuAAC samples, incubated for 5 min at RT with shaking (1000 rpm). Absolute ethanol (400 μL) was added to each sample, and the samples were incubated for 5 min at RT with shaking (1000 rpm). Samples were then placed on a magnetic rack, washed three times with 80% ethanol in water (400 μL). After washing, beads were resuspended in 200 μL 2 M urea in 0.5% SDS/PBS. DTT (10 μL of 200 mM stock in water, final concentration = 10 mM) was added into each sample and the sample was incubated at 65 °C for 15 min. Then, iodoacetamide (10 μL of 400 mM stock in water, final concentration = 20 mM) was added and the solution was incubated for 30 min at 37 °C with shaking in the dark. Absolute ethanol (400 μL) was added to each sample, and the samples were incubated for a further 5 min at RT with shaking (1000 rpm). Beads were washed three times with 80% ethanol in water (400 μL). Next, beads were resuspended in 200 μL 2 M urea in PBS and 2 μL trypsin solution (Worthington Biochemical, LS003740, 1 mg/mL in 666 μL of 50 mM acetic acid and 334 μL of 100 mM CaCl2) was added. Digest was overnight at 37 ºC with shaking. After digestion, ~ 4 mL acetonitrile (> 95% of the final volume) was added to each sample and the mixtures were incubated for 10 min at RT with shaking (1000 rpm). The beads were then washed three times with 1 mL acetonitrile each with a magnetic rack. Peptides were

eluted from SP3 beads with 50 µL of 2% DMSO in Molecular Biology Grade (MB) water for 30 min at 37 °C with shaking (1000 rpm). The elution was repeated with 50 µL of 2% DMSO in MB water. Two eluants were combined. Samples were then enriched as described below.

Proteomic sample preparation for TMT profiling of Cysteines.
Procedure adapted from previous methods for cysteine profiling with TMT.[4,5] HEK293T proteome (100 µL of 2 mg/mL) in a 1.5mL centrifugal tube was first labeled with Desthiobiotin polyethyleneoxide Iodoacetamide (**IA-DTB**)(1 µL of 50 mM stock solution in DMSO, final concentration = 500 µM) for 1h at ambient temperature. After labeling, each sample was treated with 0.5 µL benzonase (Fisher Scientific, 70- 664-3) for 30 min at 37 °C. For each 100 µL sample (1 mg/mL protein concentration), 20 µL Sera-Mag SpeedBeads Carboxyl Magnetic Beads, hydrophobic (GE Healthcare, 65152105050250) and 20 µL Sera-Mag SpeedBeads Carboxyl Magnetic Beads, hydrophilic (GE Healthcare, 45152105050250) were mixed and washed with water for three times. The bead slurries were then transferred to the IA-DTB labeled samples and incubated for 5 min at RT with shaking (1000 rpm). Absolute ethanol (400 µL) was added to each sample, and the samples were incubated for 5 min at RT with shaking (1000 rpm). Samples were then placed on a magnetic rack, washed three times with 80% ethanol in water (400 µL). After washing, beads were resuspended in 200 µL 2 M urea in 0.5% SDS/PBS. DTT (10 µL of 200 mM stock in water, final concentration = 10 mM) was added into each sample and the sample was incubated at 65 °C for 15 min. Then, iodoacetamide (10 µL of 400 mM stock in water, final concentration = 20 mM) was added and the solution was incubated for 30 min at 37 °C with shaking in the dark. Absolute ethanol (400 µL) was added to each sample, and the samples were incubated for a further 5 min at RT with shaking (1000 rpm). Beads were washed three times with 80% ethanol in water (400 µL). Next, beads were resuspended in 200 µL 2 M urea in PBS and 2 µL trypsin solution (Worthington Biochemical, LS003740, 1 mg/mL in 666 µL of 50 mM acetic acid and 334 µL of 100 mM CaCl2) was added. Digest was overnight at 37 ºC with shaking. After digestion, ~ 4 mL acetonitrile (> 95% of the final volume) was added to each sample and the mixtures were incubated for 10 min at RT with shaking (1000 rpm). The beads were then washed three times with 1 mL acetonitrile each with a magnetic rack. Peptides were eluted from SP3 beads with 50 µL of 2% DMSO in Molecular Biology Grade (MB) water for 30 min at 37 °C with shaking (1000 rpm). The elution was repeated with 50 µL of 2% DMSO in MB water. Two eluants were combined. Samples were then enriched and TMT-labeled as described below.

Proteomic sample preparation for sCIP-TMT profiling of peptide N-termini.
Adapted from a previous method for n-terminal profiling of peptides,[6] HEK293T proteome (2 mg/mL) in 2M urea/PBS was digested overnight at 37°C with shaking by addition of 2 µL trypsin solution (Worthington Biochemical, LS003740, 1 mg/mL in 666 µL of 50 mM acetic acid and 334 µL of 100 mM CaCl2) was added. 600µL of 2mg/mL digested lysates was labeled with ethynl-2-pyridinecarboxaldehyde (ethynl-2PCA) at a final concentration of 500µM for 2h at 37°C, 300rpm shaking. After labeling the peptides were transferred to six (100µL each) 1.5mL centrifuge tubes. CuAAC was performed with the pre-formed sCIP-TMT capture reagent (Using TMT126, 127N, 128N, 129N, 130N, and 131; final concentration = 1 mM), TCEP (2 µL of fresh 50 mM stock in water, final concentration = 1 mM), TBTA (6 µL of 1.7 mM stock in DMSO/t-butanol 1:4, final concentration = 100 µM), CuSO4 (2 µL of 50 mM stock in water, final concentration = 1 mM), and 0.2% SDS for 1h at ambient temperature. After CuAAC labeling, samples were then combined in a 1:5:10:15:10:5 ratio and the combined sample was treated with DTT (final concentration = 10 mM) at ambient temperature for 20 min. Then, iodoacetamide (final concentration = 20 mM) was added and the solution was incubated for 30 min at ambient temperature with shaking in the dark. For each 100 µL sample (1 mg/mL peptide concentration), 20 µL Sera-Mag SpeedBeads

Carboxyl Magnetic Beads, hydrophobic (GE Healthcare, 65152105050250) and 20 µL Sera-Mag SpeedBeads Carboxyl Magnetic Beads, hydrophilic (GE Healthcare, 45152105050250) were mixed and washed with water for three times. The bead slurries were then transferred to the CuAAC samples, incubated for 5 min at RT with shaking (1000 rpm). ~ 4 mL acetonitrile (> 95% of the final volume) was added to each sample and the mixtures were incubated for 10 min at RT with shaking (1000 rpm). The beads were then washed three times with 1 mL acetonitrile each with a magnetic rack. Peptides were eluted from SP3 beads with 50 µL of 2% DMSO in Molecular Biology Grade (MB) water for 30 min at 37 °C with shaking (1000 rpm). The elution was repeated with 50 µL of 2% DMSO in MB water. Two eluants were combined. Samples were then enriched as described below.

Proteomic sample preparation for electrophilic small-molecule screening with sCIP-TMT.
HEK293T proteome (25 µL of 2 mg/mL) in a 1.5mL centrifugal tube was first labeled with either DMSO (1µL) or one of four electrophilic small-molecules (**KB02, MC, MPP, EN300**) (500µM) for 1h at ambient temperature. Next each tube was treated with **IAA** (1 µL of 100 mM stock solution in DMSO, final concentration = 1 mM) for 1h at ambient temperature. CuAAC was performed with the pre-formed sCIP-TMT capture reagent (note compounds and their corresponding channels can be found in **Figure S6**) (final concentration = 2 mM), TCEP (1 µL of fresh 25 mM stock in water, final concentration = 1 mM), TBTA (1.5 µL of 1.7 mM stock in DMSO/t-butanol 1:4, final concentration = 100 µM), CuSO4 (1 µL of 25 mM stock in water, final concentration = 1 mM), and 0.2% SDS for 1h at ambient temperature. After CuAAC labeling all samples were combined and treated with 0.5 µL benzonase (Fisher Scientific, 70- 664-3) for 30 min at 37 °C. For each 100 µL of sample (1 mg/mL protein concentration), 20 µL Sera-Mag SpeedBeads Carboxyl Magnetic Beads, hydrophobic (GE Healthcare, 65152105050250) and 20 µL Sera-Mag SpeedBeads Carboxyl Magnetic Beads, hydrophilic (GE Healthcare, 45152105050250) were mixed and washed with water for three times. The bead slurries were then transferred to the CuAAC samples, incubated for 5 min at RT with shaking (1000 rpm). Absolute ethanol (400 µL) was added to each sample, and the samples were incubated for 5 min at RT with shaking (1000 rpm). Samples were then placed on a magnetic rack, washed three times with 80% ethanol in water (400 µL). After washing, beads were resuspended in 200 µL 2 M urea in 0.5% SDS/PBS. DTT (10 µL of 200 mM stock in water, final concentration = 10 mM) was added into each sample and the sample was incubated at 65 °C for 15 min. Then, iodoacetamide (10 µL of 400 mM stock in water, final concentration = 20 mM) was added and the solution was incubated for 30 min at 37 °C with shaking in the dark. Absolute ethanol (400 µL) was added to each sample, and the samples were incubated for a further 5 min at RT with shaking (1000 rpm). Beads were washed three times with 80% ethanol in water (400 µL). Next, beads were resuspended in 200 µL 2 M urea in PBS and 2 µL trypsin solution (Worthington Biochemical, LS003740, 1 mg/mL in 666 µL of 50 mM acetic acid and 334 µL of 100 mM CaCl2) was added. Digest was overnight at 37 ºC with shaking. After digestion, ~ 4 mL acetonitrile (> 95% of the final volume) was added to each sample and the mixtures were incubated for 10 min at RT with shaking (1000 rpm). The beads were then washed three times with 1 mL acetonitrile each with a magnetic rack. Peptides were eluted from SP3 beads with 50 µL of 2% DMSO in Molecular Biology Grade (MB) water for 30 min at 37 °C with shaking (1000 rpm). The elution was repeated with 50 µL of 2% DMSO in MB water. Two eluants were combined.

Streptavidin enrichment of sCIP-TMT labeled peptides.
For each 200µL sample of 2mg/mL cellular lysates, 50 µL of Streptavidin Agarose resin slurry (Pierce, 20353) was washed one time in 8 mL PBS and then resuspended in 500 µL PBS. Peptide solutions eluted from SP3 beads were then transferred to the Streptavidin Agarose resin suspension, and the samples were rotated for 2h at RT. After incubation, the beads were pelleted by centrifugation (5,000 g, 1 min) and washed twice with 1 mL PBS each and then twice with 1

mL water each. Bound peptides were eluted via acidic cleavage of the DADPS linkage using 200µL of 2% formic acid in MB water for 30 min at ambient temperature. The elution was repeated once more with 80% acetonitrile in MB water for 2 min at ambient temperature. The combined eluants were dried (SpeedVac), then reconstituted with 5% acetonitrile and 1% FA in MB water and analyzed by LC-MS/MS.

Streptavidin enrichment of IA-DTB labeled peptides.

For each 200µL sample of 2mg/mL cellular lysates, 50 µL of Streptavidin Agarose resin slurry (Pierce, 20353) was washed one time in 8 mL PBS and then resuspended in 500 µL PBS. Peptide solutions eluted from SP3 beads were then transferred to the Streptavidin Agarose resin suspension, and the samples were rotated for 2h at RT. After incubation, the beads were pelleted by centrifugation (5,000 g, 1 min) and washed twice with 1 mL PBS each and then twice with 1 mL water each. Bound peptides were eluted using 500µL of 0.1% trifluoroacetic acid in 50% acetonitrile in MB water for 30 min at ambient temperature. The elution was repeated once more with 50% acetonitrile in MB water for 2 min at ambient temperature. The combined eluants were dried (SpeedVac), then reconstituted with 5% acetonitrile and 1% FA in MB water and analyzed by LC-MS/MS.

TMT tag labeling

Streptavidin eluants were dried (SpeedVac), resuspended in 100mM triethylammonium bicarbonate (TEAB) (ThermoFisher Scientific, 90114), and their concentrations determined using a Pierce™ quantitative peptide concentration assay (ThermoFisher Scientific, 23275). The samples were then labeled with an 6:1 ratio (µg:µg) of TMT reagent (Using TMT126 and TMT127N from the TMT10 reagent set from ThermoFisher Scientific, 90113) to peptide for 1 hour at RT followed by quenching with 5% hydroxylamine (Fisher, AAA1256014). Samples were then acidified with 5% formic acid and cleaned up using Pierce C18 tips (100 µL bed, Fisher PI87784). Samples were then dried (SpeedVac), then reconstituted with 5% acetonitrile and 1% FA in MB water. The TMT labeled peptides for were then combined in the specified ratios.

Protein expression and purification

The sequence encoding CRKL (residue numbers 1-303) was subcloned into the pET-22b(+) with a c-terminal hexa his-tag. Point mutations (C249A) were created by site-directed mutagenesis. Plasmids were propagated in TOP10 chemically competent cells. Single colonies from TOP10 grown cells were collected in 5 mL of LB supplemented with 100 µg/ml ampicillin and grown overnight (16h). Cells were harvested the following day and subjected to Zyppy Plasmid Miniprep following the manufacturer's protocol (Zymo Research, D4037). Following sequencing of plasmids, validated plasmids were transformed to BL21(DE3)pLysS e.coli cells. Single colonies were picked from an LB agar plate and grown in 10 mL of LB media supplemented with 100 µg/mL ampicillin. The cell culture was then transferred and grown in 1 L of Miller LB medium at 37 °C to an optical density (OD600) of 0.5-1.0. The culture was then cooled to 18 °C, induced with 1 mM isopropyl-β-D- 43 galactopyranoside (IPTG), and incubated for an additional 16h at 18 °C. The cells were centrifuged at 8,000 x rpm for 45 min minutes and the cell pellet was measured. The cells were resuspended in 10 mL per 1 g of cells in lysis buffer (100 mM Tris pH 7.5, 100 mM NaCl, 25 mM Imidazole). The resuspended cells were passed through a microfluidizer (Avestin Emulsiflex C3 Homogenizer; 8,000 psi x2 rounds) to ensure lysis. The cell debris was removed by centrifugation (20,000 x g, 45 min) and the supernatant was resuspended with 1 mL of Hispur Ni-NTA agarose resin (Thermo ScientificTM, PI88222). The sample was washed with two rounds of lysis buffer (2 x 50 mL). His-tagged CRKL was eluted from the resin using an elution buffer with high imidazole concentration (100 mM Tris pH 7.5, 100 mM NaCl, 250 mM Imidazole). The eluted sample was concentrated (Amicon Ultra Centrifugal Filter Unit, 4 mL 10 kDa, Fisher Scientific, UFC801024) and buffer exchanged via PD10 desalting column (Cytiva, GE17-0851-01) into storage buffer (20 mM Tris pH 7.5, 50 mM NaCl, 5 mM DTT).

Gel-based ABPP analysis

To 1 mg/ml HEK293T cell lysates was spiked in CRKL WT or CRKL C249A (final conc of 3µM). The samples (50µL per reaction) were then treated with either DMSO or compound (**KB02** or **MPP**)- at a finalnconcentration of 500 µM - for 1 hr at ambient temperature. After 1 hr the samples were split into 2 sets and 1 set (25µL) was incubated with iodoacetamide rhodamine (5µM) for 20min at ambient temperature. Next, 10uL (per 25µL sample) of loading dye was added and the samples were resolved by SDS-PAGE (Bio-Rad 4-20% Criterion Stain-Free™) and imaged using a BioRad ChemiDoc Imaging System. Loading control was performed via staining with Coomassie brilliant blue. The gel was cut in half and the half containing samples untreated with iodoacetamide rhodamine were transferred to nitrocellulose membrane (Bio-Rad) and blocked in 5% (w/v) milk in TBS (Tris-buffered saline) for 30 minutes at room temperature. The membrane was incubated with Rabbit anti His-tag mAb (AbClonal, AE086, Lot 3522082209, used at 1:3000 dilution) in 5% (w/v) milk in TBST (Tris-buffered saline with 0.1% Tween20) overnight (14-16 hours) at 4°C then washed 3 times with TBS for 5 mins. The membrane was then incubated with IRDye® 800CW Goat anti-Rabbit Secondary Antibody (LI-COR, 926-32211, Lot D20803-10, used 1:5000 dilution) in 5% (w/v) milk in TBST for 2-3 hours at room temperature and washed 3 times with TBS. Membrane was imaged on Bio-Rad ChemiDoc

(E) Mass Spectrometry Methods

Liquid-chromatography tandem mass-spectrometry (LC-MS/MS) acquisition.

Peptide samples were analyzed by liquid chromatography tandem mass spectrometry using a Thermo Scientific™ Orbitrap Eclipse™ Tribrid™ mass spectrometer or coupled with a High Field Asymmetric Waveform Ion Mobility Spectrometry (FAIMS) Interface via injection of 400-800 ng peptide per sample. Peptides were fractionated S21 online using a 18cm long, 100 µM inner diameter (ID) fused silica capillary packed in-house with bulk C18 reversed phase resin (particle size, 1.9 µm; pore size, 100 Å; Dr. Maisch GmbH). The 70 and 180-minute water-acetonitrile gradient was delivered using a Thermo Scientific™ EASY-nLC™ 1200 system at different flow rates (Buffer A: water with 3% DMSO and 0.1% formic acid and Buffer B: 80% acetonitrile with 3% DMSO and 0.1% formic acid). The detailed 70-minute gradient includes 0 – 5 min from 3 % to 10 % at 300 nL/min, 5 – 64 min from 10 % to 50 % at 220 nL/min, and 64 – 70 min from 50 % to 95 % at 250 nL/min buffer B in buffer A. The detailed 180-minute gradient includes 0 – 5 min from 2 % to 6 % at 300 nL/min, 5 – 151 min from 6 % to 50 % at 220 nL/min, and 151 – 180 min from 50 % to 95 % at 250 nL/min buffer B in buffer A. Data was collected with charge exclusion (1, 8,>8). Data was acquired using a Data-Dependent Acquisition (DDA) method consisting of a full MS1 scan (Resolution = 120,000) followed by sequential MS2 scans (Resolution varied by experiment) to utilize the remainder of the 1 second cycle time. Precursor isolation window and normalized collision energy were set as described in the study. Conditions of Liquid-chromatography (LC) Parameter Condition Column 100 µM ID fused silica capillary packed in-house with bulk C18 reversed phase resin (particle size, 1.9 µm; pore size, 100 Å; Dr. Maisch GmbH) Mass spectrometry methods used for samples from each figure are described in **Table S6**.

Table S2. Mass spectrometry methods for samples from specified figures.

Figure	Peptide Injected	Gradient	MS^1 Resolving Power	MS^2 Resolving Power	MS^3 Resolving Power	MS^2 Collision Energy	MS^3 Collision Energy	MS^2 Isolation Window (m/z)	FAIMS voltage	Scan Time (s)
3	500ng	70 Min	120K	15K	-	25-45% HCD	-	1.6	-	1
4A,C	800ng	180 Min	120K	60K	-	36% HCD	-	0.5	-35,-45,-55V	1 each voltage (3 total)
4D	400ng	180 Min	120K	-	50K	35% CID	65% HCD	0.5	-	3
5	1µg	180 Min	120K	60K	-	36% HCD	-	0.7	-40,-60, -80V	1 each voltage (3 total)
4E-F, S8	1µg	180min	60K	60K	-	36% HCD	-	0.7	-35, -45, -60	12scans for -35, and -45V, 6scans for -60V
S16	500ng	180min	120K	60K	-	36% HCD	-	0.7	-40,-60, -80V	1 each voltage (3 total)

Table S3. Files in Proteomics Identification Database (PRIDE) datasets.

Figure	File name	Experiment

3, S3(HEK293T)	NBI-B-127-25CE<br>NBI-B-127-30CE<br>NBI-B-127-35CE<br>NBI-B-127-40CE<br>NBI-B-127-45CE	Collision energy ramping experiment for IAA labeled lysates clicked to sCIP-TMTzero
4, S6, S7 (HEK293T)	NBI-B-125K<br>NBI-B-125L<br>NBI-B-125M<br>NBI-B-125N<br>NBI-B-125O<br>NBI-B-125P<br>NBI-B-125K-MS3<br>NBI-B-125L-MS3<br>NBI-B-125M-MS3<br>NBI-B-125N-MS3<br>NBI-B-125O-MS3<br>NBI-B-125P-MS3	1:1 and 1:5:10:15 mixture sCIP-TMT[10] reagents clicked to IAA labeled lysates and enriched with streptavidin. Analyzed using both MS2-FAIMS and SPS-MS3
5, S11-17 (HEK293T)	NBI-B-130A<br>NBI-B-130B<br>NBI-B-130C	Electrophilic compound screening experiment using vehicle (DMSO), MPP, MC, KB02, and EN300 in duplicate channels. Experiment performed in triplicate.
S16	NBI-B-176G_1<br>NBI-B-176G_2	Peptide n-termini labeled with ethynyl-2PCA, clicked to six unique sCIP-TMT10 reagents and combined in a 1:5:10:15:10:5 ratio. Analyzed using HRMS2-FAIMS. Experiment performed in duplicate
4E-F, S8	NBI-B-179A<br>NBI-B-179B<br>NBI-B-179C<br>NBI-B-179D<br>NBI-B-179E<br>NBI-B-179N	1:1 and 1:4 mixture sCIP-TMT[10] reagents clicked to IAA labeled lysates and enriched with streptavidin (A-E). IA-DTB labeled lysates, enriched with streptavidin, labeled with TMT126 and TMT127N mixed in a 1:1 and 1:4 ratio (N-O,Q-R).Analyzed using

	NBI-B-179O<br>NBI-B-179Q<br>NBI-B-179R	HRMS2-FAIMS. Experiment performed in duplicate

Protein and peptide identification.
Raw data collected by LC-MS/MS were searched with MSFragger (v3.7 and v3.8) and FragPipe (v19.0-19.2 and v20.0). For closed search, the "default" proteomic workflow was loaded in FragPipe and these default values were used for all settings, except as noted below. Precursor and fragment mass tolerance was set as 20 ppm. Missed cleavages were allowed up to 1. A human protein database was downloaded from UniProtKB on [January 1st, 2020] using FragPipe, containing reviewed sequences and common contaminants, with 37110 total entries. Digestion was performed in MSFragger using the 'stricttrypsin' (i.e. allowing cleavage before P) setting, peptide length was set 7 - 50, and peptide mass range was set 500 - 5000. Cysteine residues were searched with differential modifications as described in the study, allowing a max of 2 per peptide. Cys carbamidomethylation was additionally set as a variable modification (max 2 per peptide). For labile search, a single modification mass was set as a mass offset and was restricted to cysteines. Labile search mode was enabled with Y ion masses and diagnostic fragment masses set as in Figure 2 and Figure S4 for different proteomic samples, and diagnostic ion minimum intensity of 0.02. PTM-Shepherd was enabled for fragment analysis. PSM validation, protein inference, and FDR filtering were performed in PeptideProphet, ProteinProphet, and Philosopher, respectively, in FragPipe using default settings. Results were generated using the TMT integrator output where the best PSM was set to false, allow unlabeled was set to true, and the mod tag was set to the mass of the intact cysteine modification C(638.40614) for sCIP-TMT. For IA-DTB and TMT labeled samples a variable cysteine modification in MSFragger was set at C(455.2743) for IA-DTB and fixed modifications were set at K(229.1629) and N-Term peptide(229.1629) for TMT. In TMT integrator the samples were searched as above with the mod tag set to the mass of the IA-DTB modification C(455.2743). For ethynyl-2PCA labeled samples a fixed modification in MSFragger was set at N-term peptide(614.3492) and the mod tag in TMT integrator was set to the mass of the intact modification N-term(614.3492). Data are available via ProteomeXchange with identifier PXD049154.

Data analysis, processing, and visualization.
Frequency distribution and intensity of the fragment ions and peptide remainder ions were calculated based on the output of PTM-Shepherd as mean of all replicates (See supplementary data tables). Mean of the number of PSMs and peptides of all replicates were reported as bar plots. Spectra were visualized using the proteomics data viewer integration in FragPipe, FragPipe-PDV. For choosing the PSM for quantitation, TMT-integrator selects the best PSM intensity for each cysteine, being the most intense sum across all reporter channels.

Data compilation and statistics.
Custom python scripts were implemented to compile peptide_label_quant.tsv outputs from FragPipe to count unique quantified cysteines. Unique cysteines and unique peptide-spectrum matches (PSMs) were quantified for each dataset using unique identifiers consisting of a UniProt protein ID and the amino acid number of the modified cysteine. Unique proteins were established based on UniProt protein IDs. Residue numbers were found by aligning the peptide sequence to the corresponding UniProt ID protein sequence specified by FragPipe outputs. For isobaric

quantitation, ratios were calculated by taking the average of intensities in the control channels and dividing by the average intensities in the compound channels. Students' t-tests and simple linear regression analyses were performed on GraphPad Prism v9.4.1 Compound labeling data in Table S5 was manually processed to exclude cysteines with ratios in only one of the three replicates.

(F) NMR Spectra

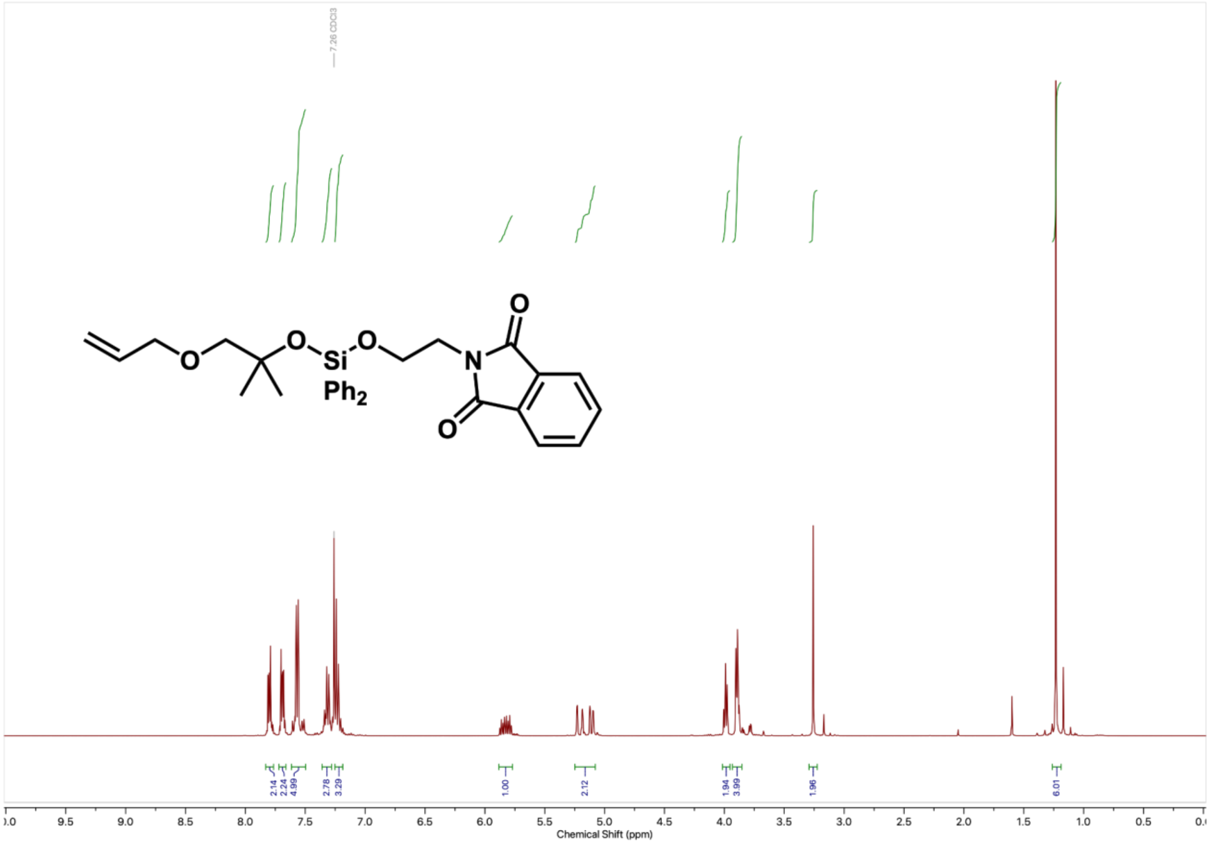

^{1}H NMR (400 MHz, $CDCl_3$) δ 7.83 – 7.76 (m, 2H), 7.73 – 7.66 (m, 2H), 7.62 – 7.50 (m, 5H), 7.35 – 7.28 (m, 2H), 7.25 – 7.18 (m, 3H), 5.83 (ddt, J = 17.3, 10.7, 5.5 Hz, 1H), 5.24 – 5.09 (m, 2H), 4.02 – 3.97 (m, 2H), 3.92 – 3.86 (m, 4H), 3.26 (s, 2H), 1.23 (s, 6H).

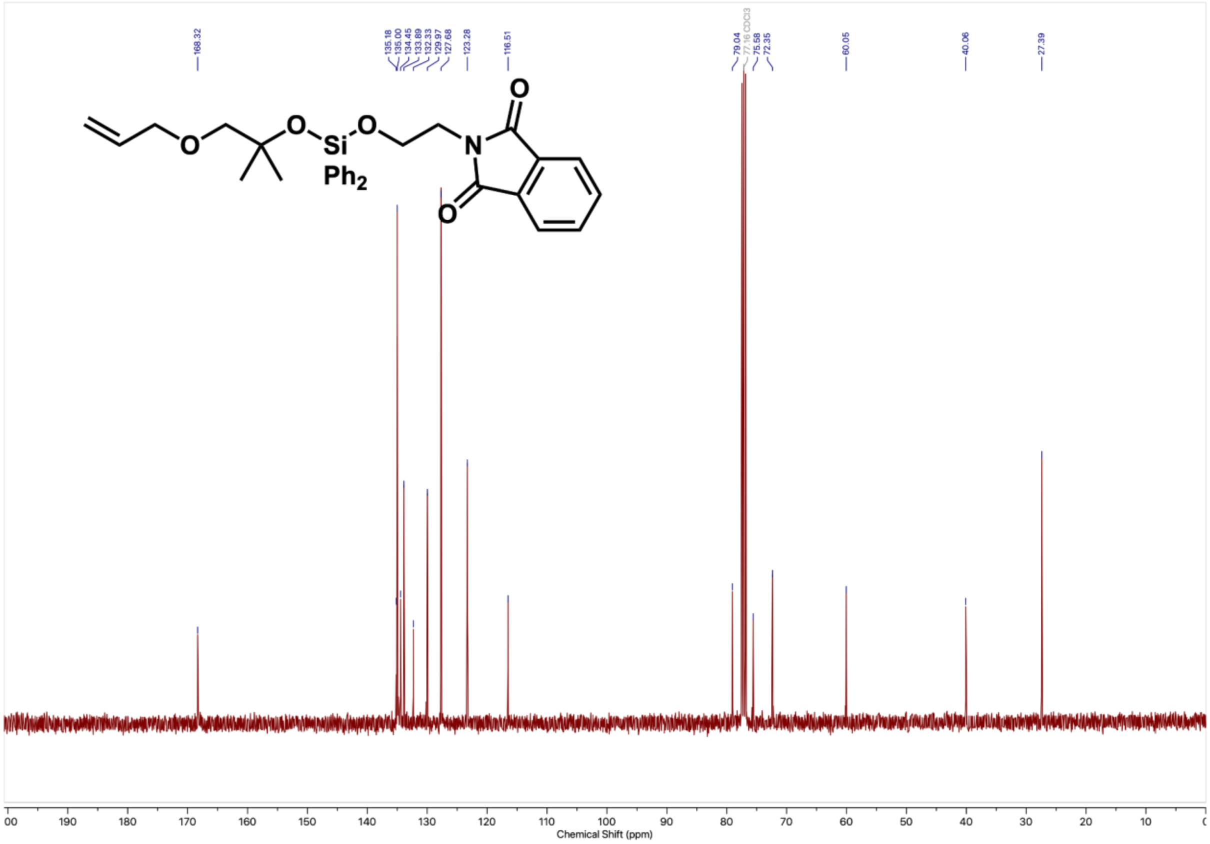

^{13}C NMR (101 MHz, $CDCl_3$) δ 168.3, 135.2, 135.0, 134.4, 133.9, 132.3, 129.9, 127.6, 123.2, 116.5, 79.0, 75.5, 72.3, 60.0, 40.0, 27.3.

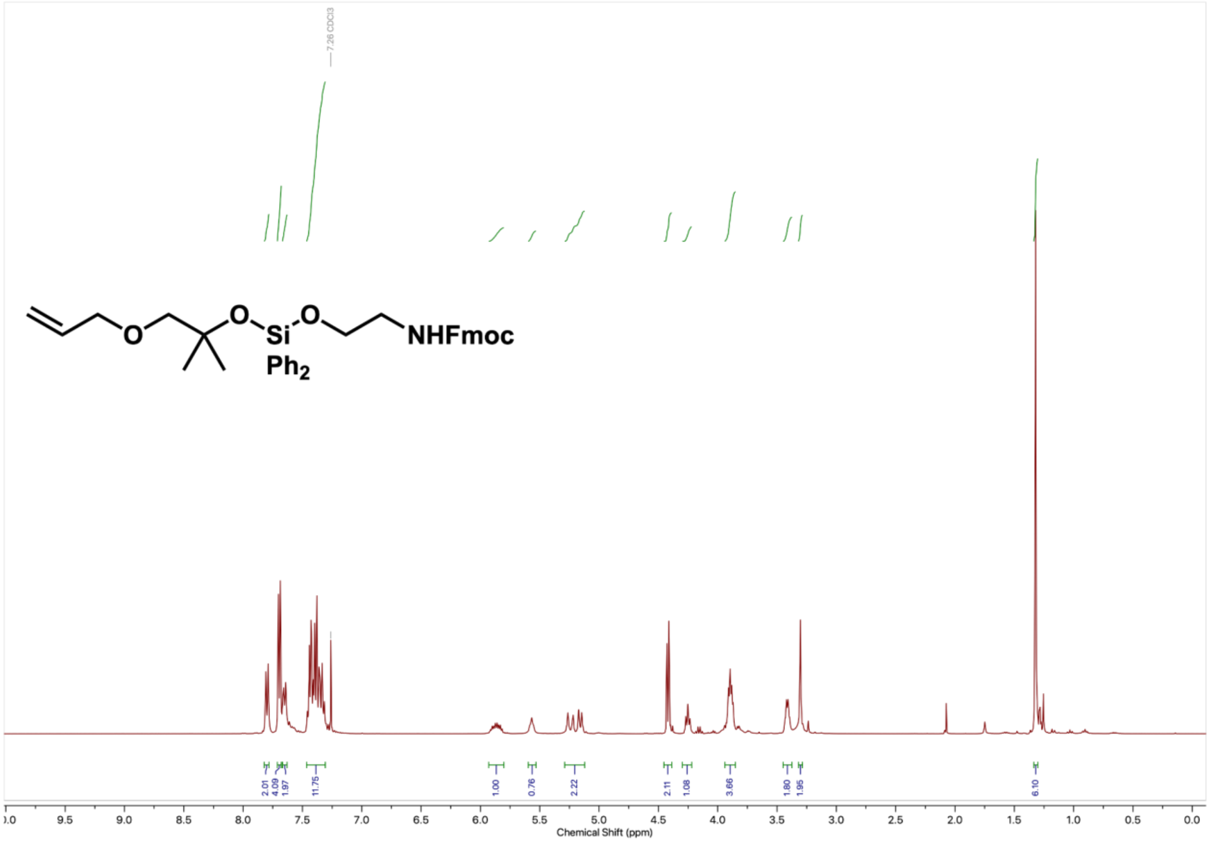

^{1}H NMR (400 MHz, $CDCl_3$) δ 7.80 (d, J = 7.6 Hz, 2H), 7.69 (dt, J = 6.7, 1.5 Hz, 4H), 7.67 – 7.63 (m, 2H), 7.47 – 7.29 (m, 10H), 5.87 (ddt, J = 16.3, 10.7, 5.6 Hz, 1H), 5.56 (d, J = 5.9 Hz, 1H), 5.29 – 5.12 (m, 2H), 4.42 (d, J = 6.9 Hz, 2H), 4.25 (t, J = 6.9 Hz, 1H), 3.94 – 3.85 (m, 4H), 3.41 (q, J = 5.3 Hz, 2H), 3.30 (s, 2H), 1.32 (s, 6H).

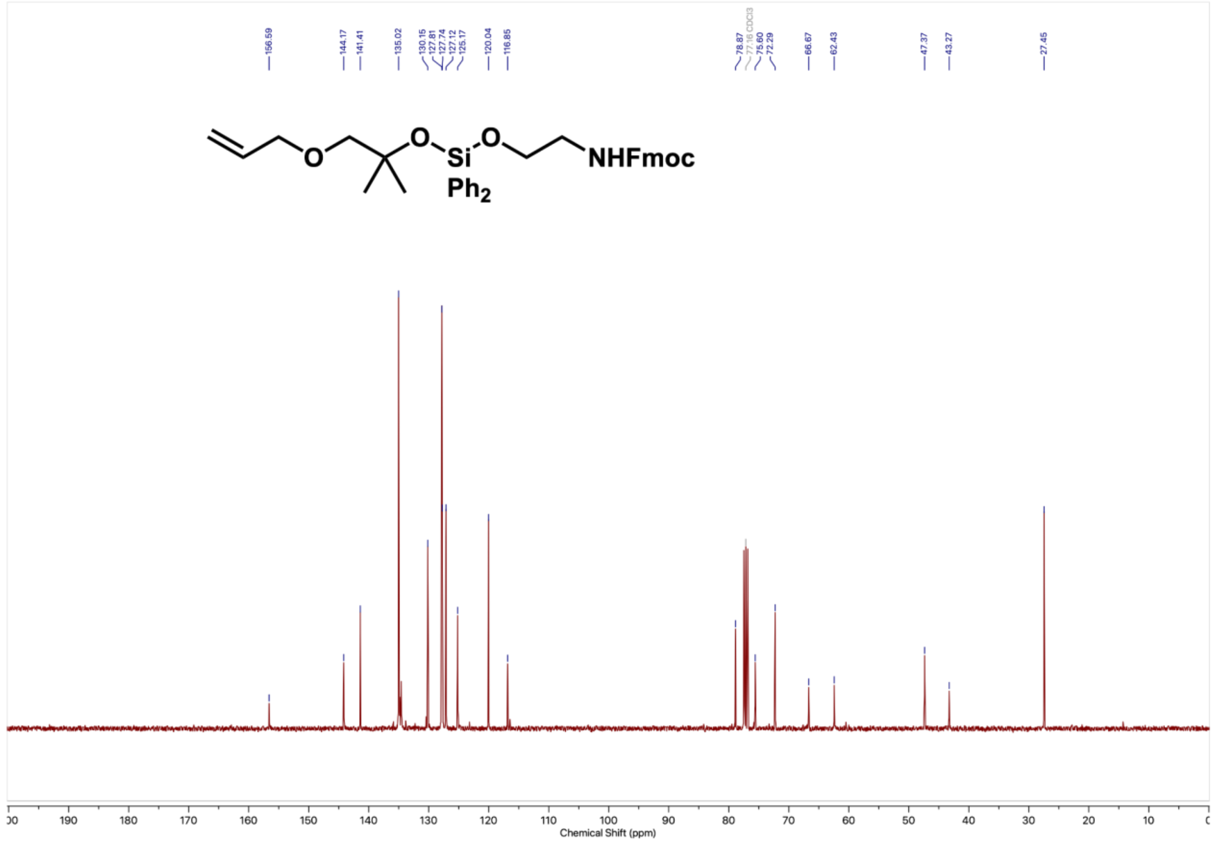

^{13}C NMR (101 MHz, $CDCl_3$) δ 156.5, 144.1, 141.4, 135.0, 130.1, 127.8, 127.7, 127.1, 125.1, 120.0, 116.8, 78.8, 75.6, 72.2, 66.6, 62.4, 47.3, 43.2, 27.4.

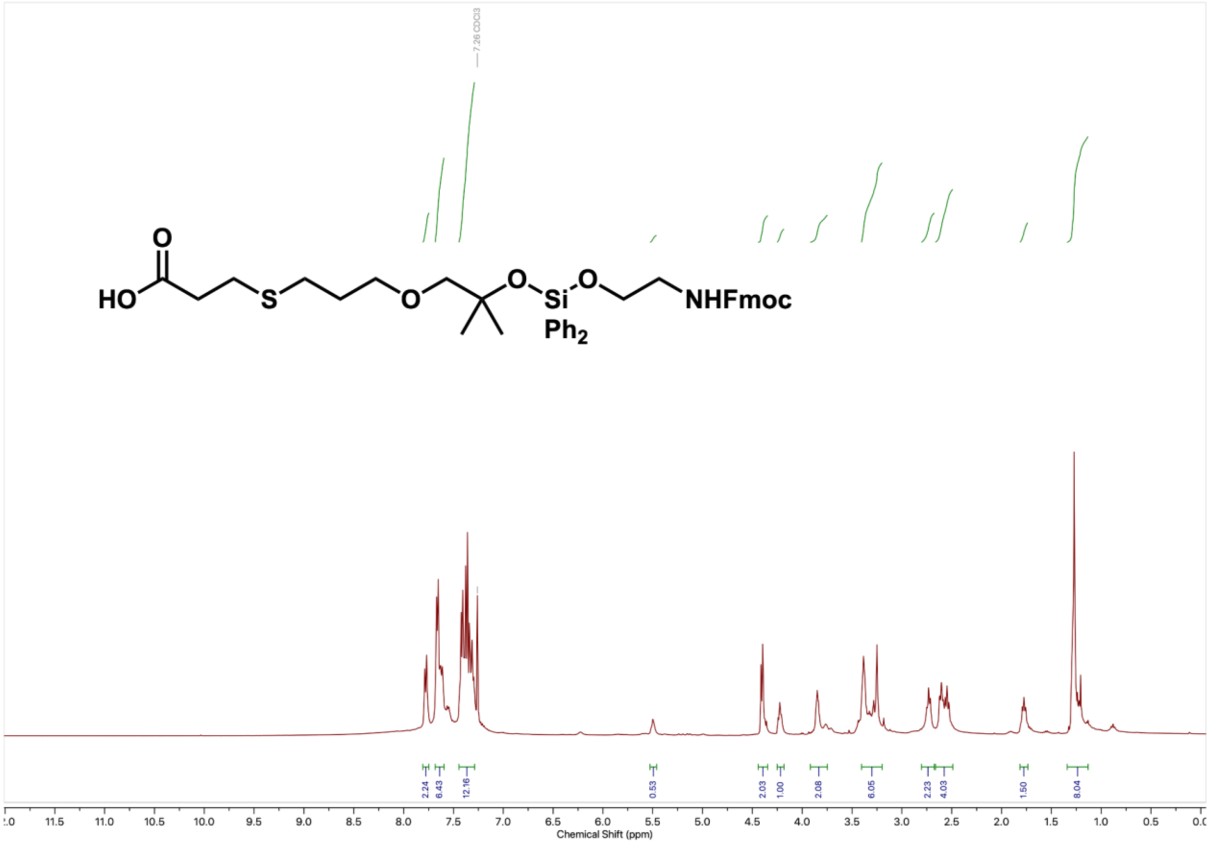

^{1}H NMR (400 MHz, $CDCl_3$) δ 7.78 (d, *J* = 7.6 Hz, 2H), 7.64 (dd, *J* = 19.6, 4.5 Hz, 6H), 7.37 (ddt, *J* = 25.2, 14.3, 5.2 Hz, 10H), 4.41 (d, *J* = 6.9 Hz, 2H), 4.23 (t, *J* = 6.8 Hz, 1H), 3.90 – 3.69 (m, 2H), 3.46 – 3.16 (m, 6H), 2.73 (t, *J* = 7.2 Hz, 2H), 2.57 (dt, *J* = 22.3, 7.3 Hz, 4H), 1.77 (t, *J* = 6.9 Hz, 1H), 1.27 (m, 8H).

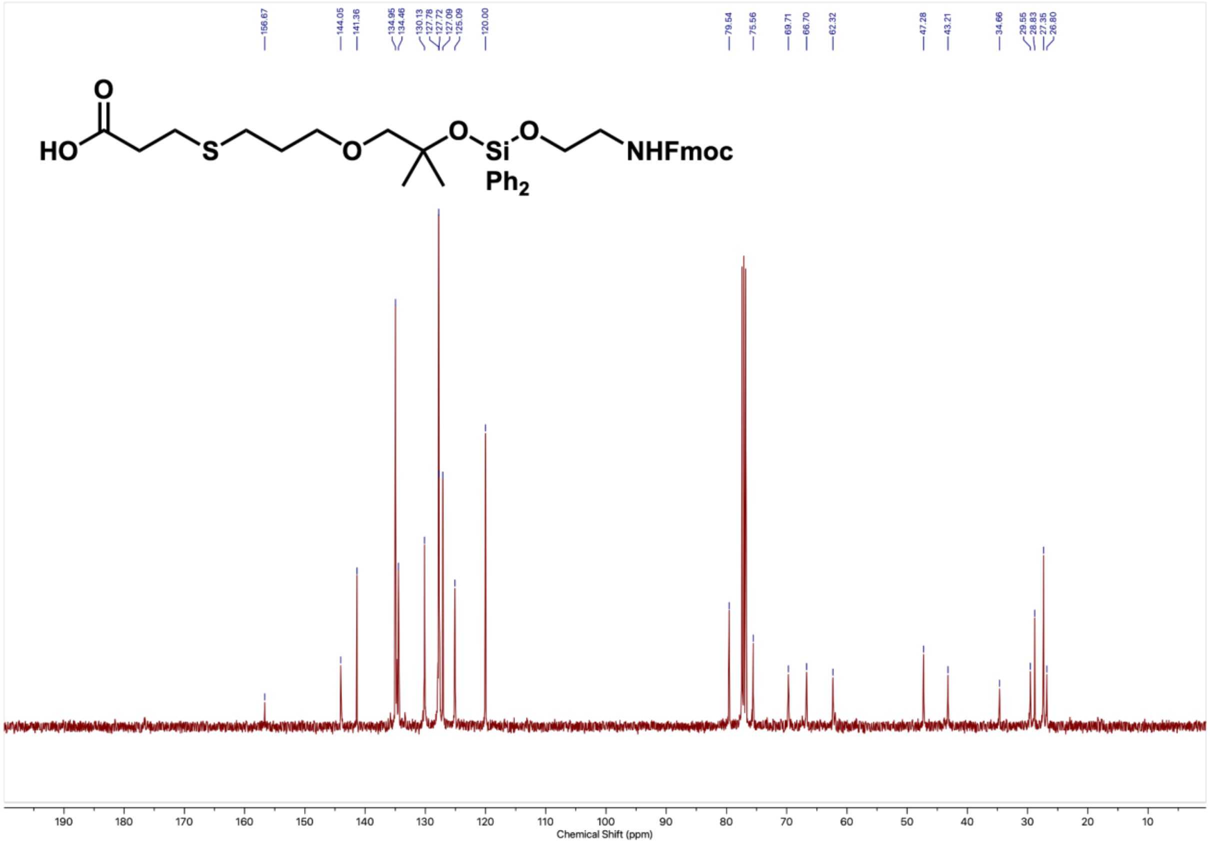

^{13}C NMR (101 MHz, $CDCl_3$) δ 156.6, 144.0, 141.3, 134.9, 134.4, 130.1, 127.7, 127.7, 127.0, 125.0, 120.0, 79.5, 75.5, 69.7, 66.7, 62.3, 47.2, 43.2, 34.6, 29.5, 28.8, 27.3, 26.8.

(G) Supplementary References

1. Burton, N. R. *et al.* Solid-Phase Compatible Silane-Based Cleavable Linker Enables Custom Isobaric Quantitative Chemoproteomics. *J. Am. Chem. Soc.* **145**, 21303–21318 (2023).

2. Boatner, L. M., Palafox, M. F., Schweppe, D. K. & Backus, K. M. CysDB: a human cysteine database based on experimental quantitative chemoproteomics. *Cell Chem. Biol.* **30**, 683-698.e3 (2023).

3. Piergentili, A. *et al.* Properly substituted 1,4-dioxane nucleus favours the selective M3 muscarinic receptor activation. *Bioorganic Med. Chem.* **17**, 8174–8185 (2009).

4. Vinogradova, E. V. *et al.* An Activity-Guided Map of Electrophile-Cysteine Interactions in Primary Human T Cells. *Cell* **182**, 1009-1026.e29 (2020).

5. Kuljanin, M. *et al.* Reimagining high-throughput profiling of reactive cysteines for cell-based screening of large electrophile libraries. *Nat. Biotechnol.* **39**, 630–641 (2021).

6. Bridge, H. N., Leiter, W., Frazier, C. L. & Weeks, A. M. An N terminomics toolbox combining 2-pyridinecarboxaldehyde probes and click chemistry for profiling protease

specificity. *Cell Chem. Biol.* (2023) doi:10.1016/j.chembiol.2023.09.009.
