# Supplementary material for: Functionalizing tandem mass tags for streamlining click-based quantitative chemoproteomics
Source: Commun Chem. 2024 Apr 10;7:80. doi: 10.1038/s42004-024-01162-x (PMC11006884; doi:10.1038/s42004-024-01162-x)
Supplement: Supplementary file 3 — Description of Additional Supplementary Files [file 42004_2024_1162_MOESM3_ESM.pdf]

Description of Additional Supplementary Files

File name: Supplementary Data 1

Description: Proteomic data related to Figure 3, defining the acquisition parameters for sCIP-TMT.

File name: Supplementary Data 2

Description: Proteomic data related Figure 4. sCIP-TMT faithfully quantifies cysteine ratios with decreased sample preparation times.

File name: Supplementary Data 3

Description: Proteomic data related to Figure 4 and Figure S9, Comparison of sCIP-TMT and TMT based sample preparation.

File name: Supplementary Data 4

Description: Proteomic data related to Figure 5, sCIP-TMT is compatible with small-molecule electrophile screening.
